# *Listeria monocytogenes* Differential Transcriptome Analysis Reveals Temperature-Dependent Agr Regulation and Suggests Overlaps with Other Regulons

**DOI:** 10.1371/journal.pone.0043154

**Published:** 2012-09-14

**Authors:** Dominique Garmyn, Yoann Augagneur, Laurent Gal, Anne-Laure Vivant, Pascal Piveteau

**Affiliations:** 1 Université de Bourgogne, UMR1347, Dijon, France; 2 INRA, UMR 1347, Dijon, France; 3 AgroSup Dijon, UMR1347, Dijon, France; National Jewish Health and University of Colorado School of Medicine, United States of America

## Abstract

*Listeria monocytogenes* is a ubiquitous, opportunistic pathogenic organism. Environmental adaptation requires constant regulation of gene expression. Among transcriptional regulators, AgrA is part of an auto-induction system. Temperature is an environmental cue critical for *in vivo* adaptation. In order to investigate how temperature may affect AgrA-dependent transcription, we compared the transcriptomes of the parental strain *L. monocytogenes* EGD-e and its *ΔagrA* mutant at the saprophytic temperature of 25°C and *in vivo* temperature of 37°C. Variations of transcriptome were higher at 37°C than at 25°C. Results suggested that AgrA may be involved in the regulation of nitrogen transport, amino acids, purine and pyrimidine biosynthetic pathways and phage-related functions. Deregulations resulted in a growth advantage at 37°C, but affected salt tolerance. Finally, our results suggest overlaps with PrfA, σB, σH and CodY regulons. These overlaps may suggest that through AgrA, *Listeria monocytogenes* integrates information on its biotic environment.

## Introduction

*Listeria monocytogenes* is a Gram+bacterium characterised by a low G+C content. One of the striking features of this bacterium is the variety of its life styles [1]. Indeed, as a saprophytic bacterium, *L. monocytogenes* is detected in many habitats such as soil [2], [3], vegetation [4], water systems [5], farm environments [6], [7], food-stuff and the food processing environment [8] where the bacterium colonises abiotic surfaces and form biofilms [9]. Another facet of the ecology of this bacterium is its ability to survive in the gastrointestinal tract of animals and Humans, to multiply intracellularly and eventually to cause life-threatening diseases [10], [11].

The extensive repertoire of transport proteins and regulatory genes support the ability to colonise a wide range of ecosystems [12]. A large percentage of the genome of this ubiquitous bacterium is dedicated to regulation including 209 transcriptional regulators [12]. Amongst these, AgrA belongs to an autoinduction system organised as a four-gene operon *agrBDCA* [13], [14]. AgrC/AgrA is a typical two component system that responds to the presence of the auto-inducing peptide. Inactivation of the Agr system affects the ability of *L. monocytogenes* to form biofilms at 25°C [15] and lowers its ability to generate infection in a murine model [16].

Environmental adaptation requires integration of a variety of environmental cues that results in deep transcriptional reshaping during saprophytic [17] and *in vivo* [18] life-styles. Temperature is one of these environmental cues that *L. monocytogenes* integrates in order to regulate gene expression. Expression of virulence factors is up-regulated at 37°C [19], [20] while expression is repressed at lower temperatures. Another example is the down-regulation of mobility and chemotaxis genes at 37°C [21].

In the present paper, we investigated how temperature affects Agr-dependent regulation through a combination of physiological tests and differential transcriptome analyses during growth at 25°C and 37°C.

## Results

### *L. monocytogenes* Gene Expression Patterns in the Parental and *ΔagrA* Backgrounds are Influenced by Temperature

**Figure 1:**
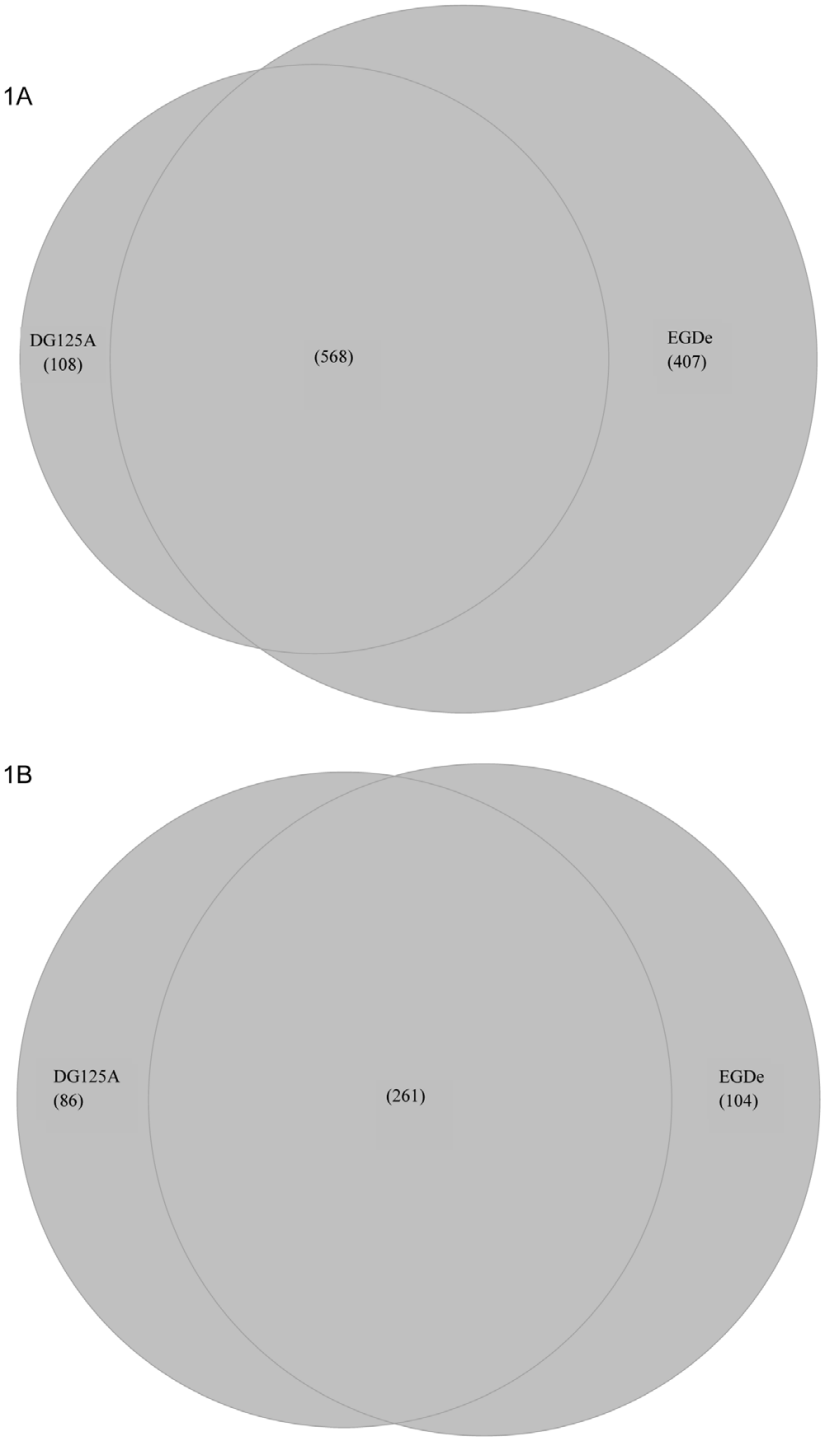
Venn diagrams of the overall number of genes with temperature-dependent variations and represented as specific or common to *L. monocytogenes* EGD-e and *L. monocytogenes* DG125A. (A) higher transcript levels at 25°C; (B) higher transcript levels at 37°C.

**Figure 2:**
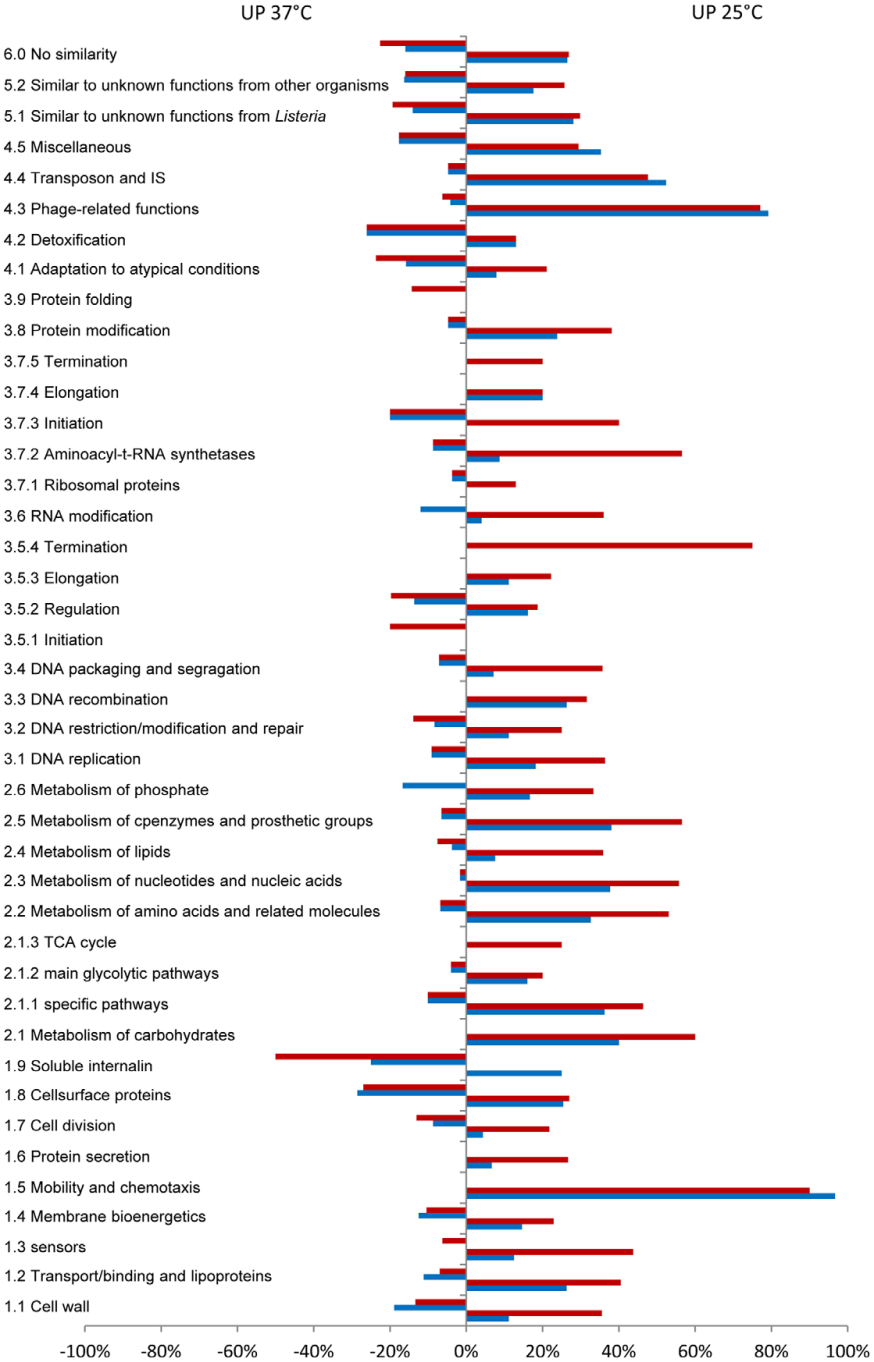
Percentage of genes varying significantly according to the temperature and distributed within functional categories. (red boxes) *L. monocytogenes* EGD-e; (blue boxes) *L. monocytogenes* DG125A.

Figure 1.A presents the sets of genes with higher transcript levels at 25°C than at 37°C in *L. monocytogenes* EGD-e and in the *ΔagrA* mutant (DG125A). Differential analysis of the level of transcripts of *L. monocytogenes* EGD-e on the basis of the growth temperature showed that 975 genes had higher transcript levels at 25°C than at 37°C (Figure 1.A). Most genes from category 1.5 (Motility and chemotaxis) and 4.3 (Phage-related genes) were represented in this group of genes (Figure 2). Similarly, several genes from categories intermediary metabolism (2.1.1, 2.2, 2.3, 2.5), transport/binding proteins (1.2), regulators (3.5.2), ribosomal proteins (3.7.1) and similar to unknown functions (5.2, 6) were most likely to be transcribed to higher levels at 25°C as indicated by gene ontology. Among these, a set of 568 genes was common to EGD-e and DG125A.

The variation of transcripts of 407 genes was specific to the EGD-e background (Figure 1A.). The levels of transcripts of these genes were significantly higher at 25°C (Table S1). Gene ontology identified the functional categories 1.1 (cell wall), 2.2 (Metabolism of amino acids and related molecules), 2.4 (metabolism of lipids) and 3.7.2 (Aminoacyl-tRNA synthetases) as significant terms (Figure 2). For example *dltB* is involved in D-alanine esterification of teichoic acid and deletion of the *dlt* operon affects adhesion to cell lines and virulence [22]. *lmo1076* encodes AUTO an autolysin required for virulence [23], [24], proteins encoded by *mreB* and *mreC* are involved in cell-shape determination [25], proteins encoded by *tagB*, *tagD* are involved in wall teichoic acid synthesis [26].

Finally, 108 genes had specifically higher transcript levels at 25°C than at 37°C in the *ΔagrA* mutant (Table S2) but functional category was not a significant term within this set of genes.

Results of the comparison of genes with higher transcript levels at 37°C are presented Figure 1B. This analysis identified a set of 365 genes in the parental strain EGD-e. Ten functional categories (1.5, 1.6, 2.1, 2.1.3, 3.3, 3.5.3, 3.5.4, 3.6, 3.7.4, 3.7.5) were not represented (Figure 2). The transcription of 39 regulators increased with temperature. Among known regulators, *agrA* [13], *zurR* [27], *codY* [28] and *prfA* [29] varied by 2.2, 2.4, 3.4 and 4.5 respectively.

In the *ΔagrA* background, transcription of 347 genes increased with temperature while a set of 261 genes was common to both backgrounds. Gene ontology identified functional categories 1.8 (Cell surface proteins), 5.2 (unknown proteins from other organisms) and 6.0 (no similarity) as significant terms in this set of genes.

A set of 104 genes varying significantly with temperature was specific to EGD-e (Table S3). Notably, the level of transcripts of 16 regulators including AgrA and PrfA were represented in this set of genes. Finally, a set of 86 genes was specific to DG125A (Table S4).

**Figure 3:**
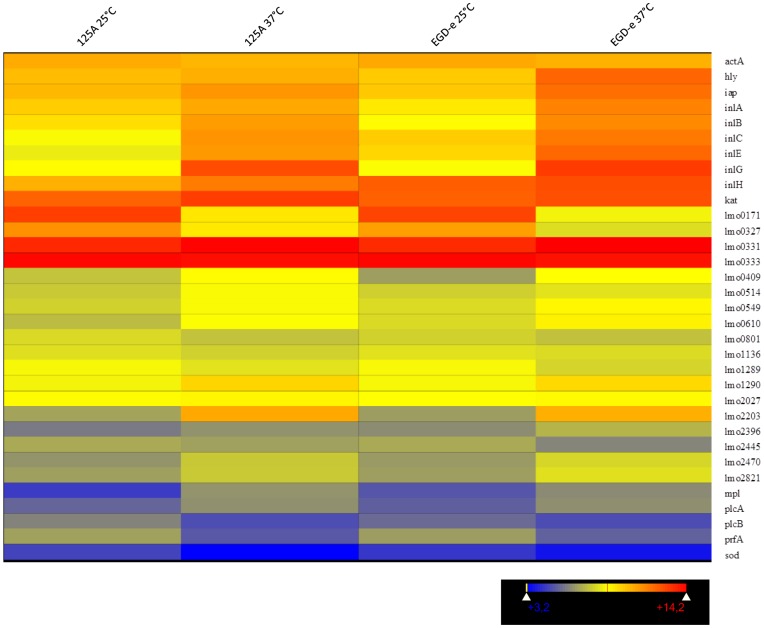
Heatmap of virulence genes.

Considering the central role of temperature in the regulation of virulence, we focused on the major virulence and internalin genes. As expected, this analysis showed that transcription of the central virulence gene cluster was higher at 37°C than at 25°C in EGD-e (Figure 3). However transcription of *prfA* and *plcA* did not significantly vary according to the growth temperature in the deletion mutant. In the EGD-e background, among internalins, *inlB*, *inlC*, *inlH*, *lmo0610*, *lmo0801*, *lmo2027*, *lmo2396* and *lmo2821* had higher transcript levels at 37°C than at 25°C. On the opposite, *lmo0327*, *lmo0514*, and *lmo2445* were transcribed to higher levels at 25°C than at 37°C. Deletion of *agrA* affected these results. Indeed in DG125A, during incubation at 37°C, higher transcription was observed for *inlA*, *inlB*, *inlH*, *lmo0331*, *lmo0333*, *lmo0610*, *lmo0801*, *lmo2027* and *lmo2821*. At 25°C, higher transcripts were detected for *lmo0327*, *lmo0514*, *lmo1289*, *lmo2445* and *lmo2470*.

### Deletion of *agrA* Results in Moderate Variations of Transcriptome at 25°C

**Figure 4:**
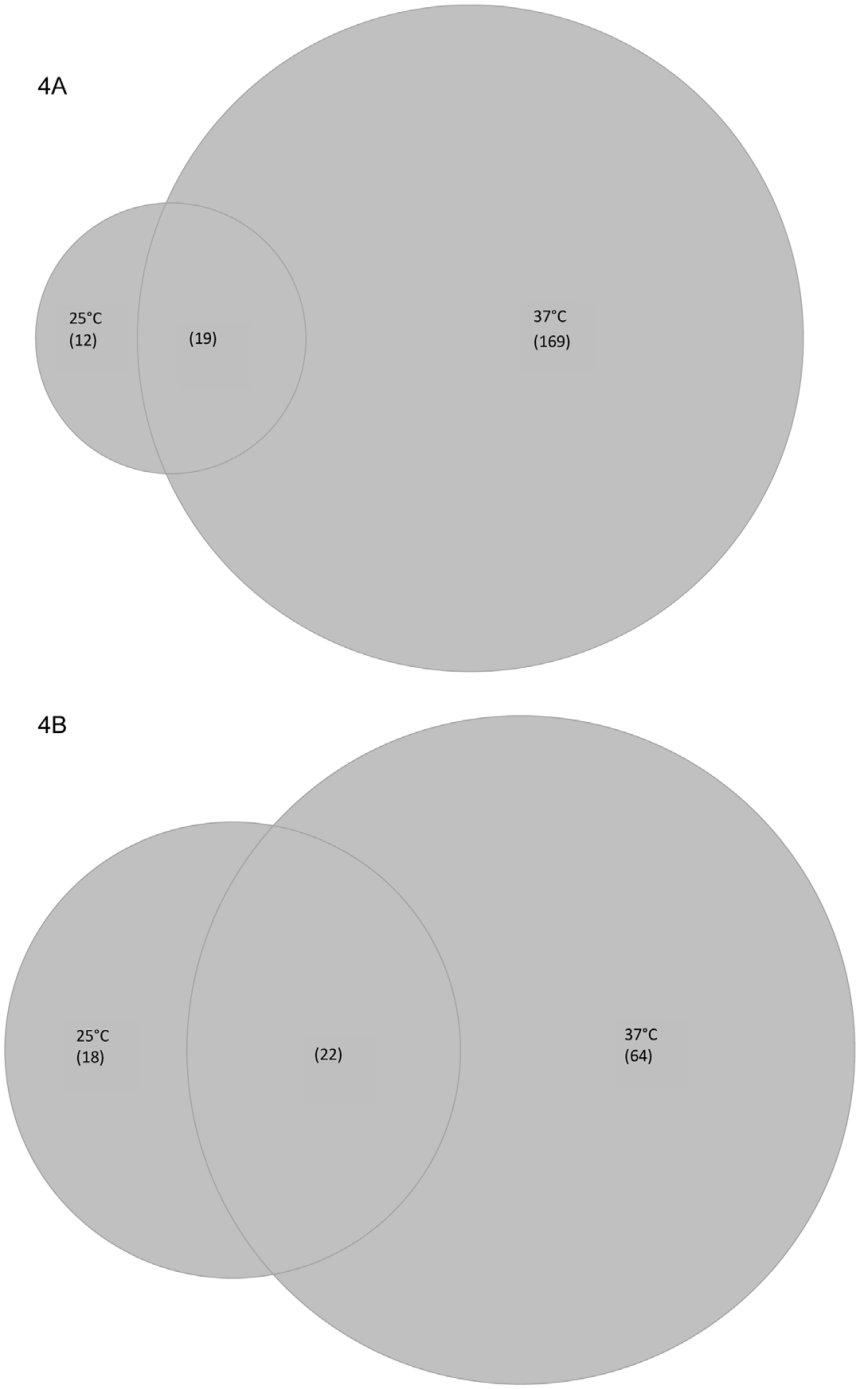
Venn diagrams of genes with significant differences in the comparison DG125A versus EGD-e at 25°C and 37°C. (A) genes with higher transcript levels; (B) genes with lower transcript levels.

In order to gain a better understanding of the regulatory role of AgrA, we analysed the differences of levels of transcripts in the mutant background compared to the parental background (DG125A vs EGD-e). At 25°C, this differential analysis showed that 31 genes had higher transcript levels in DG125A in comparison to EGD-e (Figure 4A, Tables S5, S7). The ortholog of the *ytmI* operon of *B. subtilis* encoding an L-cystine ABC transporter, a riboflavin kinase and proteins of unknown functions (*lmo2343-lmo2352*) belonged to this set of genes [30]. Other genes encoding proteins involved in amino acid transport were detected, for example *lmo0152* (similar to oligopeptide ABC transporter-binding protein). Transcription of the *nadABC* operon, involved in NAD synthesis, was also increased in DG125A. Gene ontology did not identify significant functional category terms in this set of genes.

40 genes were detected with lower transcript levels in the *ΔagrA* genetic background (Figure 4B, Tables S5, S7). As expected *agr* genes varied with fold changes as high as 36 (*agrD*). Three genes coding for putative secreted proteins (*lmo477*, *lmo478*, *lmo479*) varied by 21.4, 12.3 and 4.9 respectively. Other genes included in this set of genes were *lmo0880* (similar to wall associated protein precursor (LPXTG motif), Internalin genes *inlA*, *inlB* and *inlH* and the complete *opuCA*-*opuCD* operon. Gene ontology did not identify functional category as significant.

### Genes of Transport and Metabolism of Amino Acids and Related Molecules are Over-expressed in the *ΔagrA* Background during Growth at 37°C

**Figure 5:**
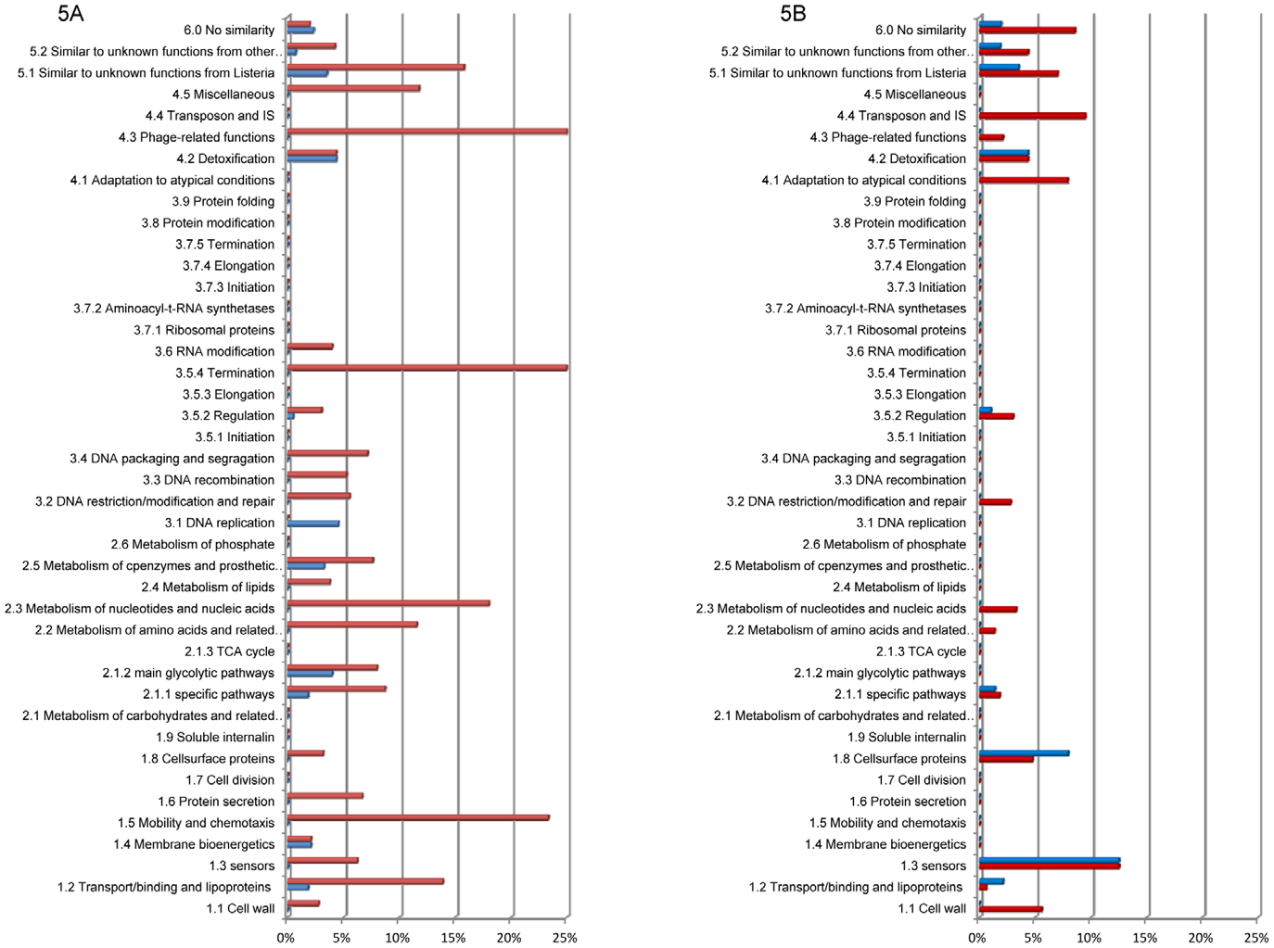
Percentage of genes varying significantly in the comparison DG125A versus EGD-e at 25°C (blue boxes) and 37°C (red boxes), distributed within functional categories. (A) genes with higher transcript levels; (B) genes with lower transcript levels.

The results at 37°C were different. 188 genes had transcript levels significantly higher in DG125A vs EGD-e (Tables S6, S7). Gene ontology showed that functional categories 1.2–4.3–6.0–1.5–5.2–2.3–2.2–5.1 were significant terms (Figure 5).

46 genes of the functional category “Transport/binding proteins and lipoproteins“ (functional category 1.2) had higher transcript levels in the *ΔagrA* mutant than in EGD-e when grown at 37°C. Glutamine, amino acids (L-cyctine, histidine, methionine) spermidine/putrescine and oligopeptides ABC transporters were included in this set of genes. Hortologs (*lmo2343* to *lmo2351*) of the *ytmI* operon of *B. subtilis* involved in L-cystine transport [30] and its transcriptional regulator *ytlI* (*lmo2352*) are represented in this set of genes and most are included in the top 20 of genes with higher transcript levels. *lmo1740*, *lmo1739* and *lmo1738*, are co-transcribed [18] and *in silico* analysis suggested it was specific to histidine [31]. The oligopeptide ABC transporters (*lmo0135 lmo0136 lmo0152*) are structurally related to OppA [32]. *lmo0135* encodes a multifunctional protein involved in cysteine transport, acid resistance and is required for virulence [33]. Finally, *lmo1516 lmo1517* are co-transcribed (operon 251 [18]) and encode an ammonium transporter which is down regulated in PrfA* backgrounds [34]. Several genes involved in PTS systems (*lmo0738*, *lmo1255*, *lmo1973*, *lmo1971*, *lmo1035*, *lmo1901*, *lmo2000*, *bvrB*) [35] and putative sugar ABC transporters (for example *lmo0768*, *lmo0767* and *lmo0766*) had higher transcript levels in DG125A compared to EGD-e. Two genes with higher transcript level (*lmo1884* and *pyrP*) encode permeases for xanthine and uracil. Both genes are co-transcribed with genes encoding enzymes that participate to purine and pyrimidine metabolism. Indeed, most genes (*pyrE pyrF pyrD pyrDII pyrAB pyrAa pyrC pyrB pyrP*) of the operon encoding components of the pyrimidine biosynthesis pathway had transcript levels higher than 2. Similarly, *lmo1885*, *lmo0132* and *purE* encoding enzymes of the purine biosynthesis pathway had higher transcript levels.

8 out of 30 “motility and chemotaxis” (functional category 1.5) genes had higher transcript levels in DG125A. *lmo0678* and *lmo0679* code for proteins similar to flagellar biosynthetic proteins FliR and FlhB. Lmo0683 is similar to chemotactic methyltransferase CheR, Lmo0697 is similar to the flagellar hook protein FlgE, Lmo0699 is similar to the flagellar switch protein FliM, Lmo0711 is similar to flagellar basal-body rod protein FlgC, and Lmo0712 is similar to the flagellar hook-basal body complex protein FliE. We did not evidence any difference in motility between EGD-e and DG125A and both strains were immobile at 37°C (data not shown).

Considering “Metabolism of amino acids and related molecules” (functional category 2.2), 17 genes had higher transcript levels including genes encoding enzymes from amino acids biosynthetic pathways, for example in the conversion of glutamine to glutamate (*lmo1733* and *lmo1734*) and in the arginine and aromatic amino acid (*lmo1926*, *hisC*, *aroB* and *tyrA*) biosynthesis pathways [36].

Interestingly, six genes encoding regulators had higher transcript levels. These included *lmo2352* the ortholog of *ytlI,* the activator of the *ytmI* operon encoding an L-cyctine ABC transporter [30], *codY* which encodes a regulator responsive to GTP and branched chain amino acids [28] and *lmo2107* coding a putative transcriptional regulator (DeoR family) up regulated during heat shock [37].

86 genes had lower transcript levels in the *ΔagrA* background (Tables S6, S7). As excepted *agr* genes varied with fold changes as high as 29 (*agrD*). Three genes coding for putative secreted proteins (*lmo477*, *lmo478*, *lmo479*) varied by 44.6, 41.9 and 43.5 respectively. Five regulators (*prfA*, *comK*, *lmo0083*, *lmo0602*, *lmo2744*) were represented in this set of genes. Over 60% of the genes of this set code for proteins of unknown functions and functional category 6 was a significant term.

### Genes Following Similar Trends in the Analysis “DG125A vs EGD-e” Regardless of Temperature

Nineteen genes had higher transcripts levels in DG125A than in EGD-e regardless of temperature (Figure 4A, Table S7). Five genes code for peptides and amino acids transporters. Four genes from intermediary metabolism, one monooxygenase and eight genes coding for unknown proteins were in this set of genes.

Transcription of 22 genes was lower in the *ΔagrA* than in the parental background. The *agr* operon, *inlA*, *inlB* and genes coding for hypothetical proteins were in the set of genes.

**Figure 6:**
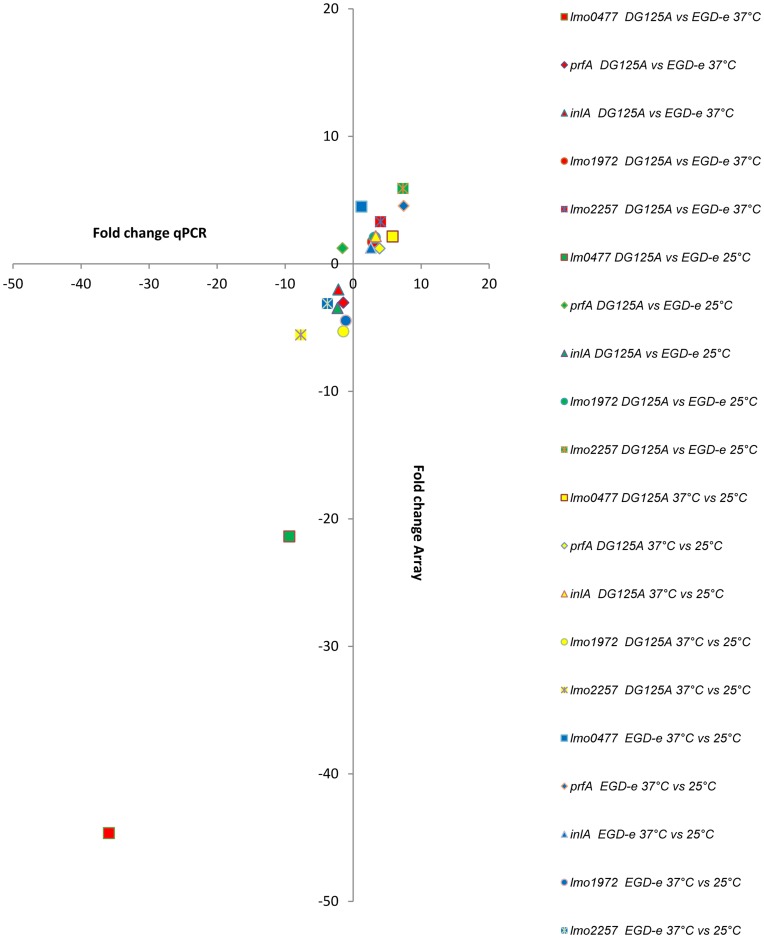
Comparison of fold-changes determined from microarray and reverse transcriptase real time PCR data. (□) *lmo0477*, (◊) *prfA*, (Δ) *inlA*, (Ο) *lmo1972*, (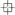) *lmo2257*; red : DG125A versus EGD-e 37°C, green : DG125A versus EGD-e 25°C, blue : EGD-e 37°C versus 25°C, yellow : DG125A 37°C versus 25°C.

The overall results observed in the comparisons between strains (DG125A versus EGD-e at 25°C and 37°C) and between temperatures (37°C versus 25°C, backgrounds EGD-e and DG125A) were confirmed by reverse transcription Real-time PCR quantification of the transcripts of a selection of five genes (*lm0477*, *prfA*, *inlA*, *lmo1972*, *lmo2257*). As shown figure 6, similar trends were observed. Furthermore, in order to confirm that these transcriptional differences were a consequence of the deletion of *agrA*, we complemented DG125A with the parental version of the gene and performed quantification of transcripts of *agrA*, *lmo0477*, *prfA* and *lmo1972.* As presented in supplementary material S1, complementation of DG125A restored the level of transcripts of this selection of genes.

### Deletion of *agrA* Affects Transcription of Sigma Factors, CodY, CtsR, HrcA, PrfA and VirR Regulons

Table 1 shows the percentage of regulons that varied in the *ΔagrA* background at 25°C and 37°C. At 25°C, the transcription of the HrcA and VirR regulons did not significantly vary and a limited number of genes of the σC, σL, σH, CodY and CtsR regulons had transcripts levels significantly different in DG125A than in EGD-e background. The proportion of variation in the σB and PrfA regulons was higher. The transcription of 18 genes under the positive control of the σB was lower. Similarly, 18 genes under the positive control of PrfA were represented in the set of genes with lower transcript levels. Interestingly, 14 of them are co-regulated by both PrfA and σB (36.8% of the genes co-regulated by PrfA and σB). Gene ontology analysis identified “co-regulation by PrfA and σB”, “PrfA”, “σB” as significant terms in the set of genes with lower transcripts levels at 25°C. None of the regulon terms was significant in the set of genes with higher transcript levels at 25°C.

**Table 1:** Number of genes from sigma factors and other transcriptional regulators regulons with significant variations of transcripts between *L. monocytogenes* DG125A and *L. monocytogenes* EGD-e at 25°C and 37°C.

Regulon	Percentage of regulon	
	25°C	37°C
σB (216)^a^	8.3* (18)^b^	12.4* (26)
σC (24)	4.2 (1)	8.3 (2)
σL (51)	3.9 (2)	7.8 (4)
σH (169)	4.1 (7)	14.8* (25)
CodY (86)	2.3 (2)	23.2* (20)
CtsR (64)	3.1 (2)	10.9 (7)
HrcA (61)	0	0
PrfA (70)	25.7* (18)	15.7* (11)
VirR (17)	0	0

* significant ontology term.

a indicated in brackets is the total number of genes in the regulon.

b indicated in brackets is the number of genes with significant variation of transcript level.

At 37°C transcription of the HrcA and VirR regulons was not affected in the mutant background. Twenty genes of the CodY regulon, under the negative control of CodY, were detected with higher transcripts levels in DG125A and “CodY” was a significant ontology term. The percentage of variation was higher in the σC, σL, σH and CtsR with 2, 4, 25, and 7 genes respectively. “σH” was a significant term in the set of genes with lower transcripts levels. All of these genes are positively regulated by σH. Regarding the σB regulon, a total of 26 genes varied. Nine of them belonged to the set of genes with higher transcript levels. These genes are under the negative control of σB except *lmo0735, lmo0736, lmo0737* and *lmo0738*. Seventeen genes under the positive control of σB were included in the set of genes with lower transcript level. Finally, 11 genes positively regulated by PrfA had lower transcripts levels in the *ΔagrA* than in the parental background at this temperature. “co-regulation by PrfA and σB”, “PrfA” and “σB” were all significant terms in this set of genes with lower transcripts levels.

### Inactivation of AgrA Improves Growth Rates of Planktonic Cultures but Reduces Salt Tolerance

As presented above, deletion of *agrA* resulted in significant differences of transcription at both temperatures; transcription of genes related to amino acids metabolism was greater in the *ΔagrA* background than in the parental strain, conversely transcription of the *opuCACD* operon was lower when AgrA was not functional. We therefore investigated whether these variations translated into phenotypic traits especially regarding the ability of the mutant to utilize amino acids as well as its salt stress tolerance. We first of all compared growth of both strains at 25°C and 37°C in the complex rich medium TSB and in the chemically defined medium KRM supplemented with casamino acids. As shown Table 2, at 25°C in the chemically defined medium supplemented with amino acids, the growth of strain DG125A was significantly faster than EGD-e but the differences were not significant during growth in the complex medium TSB. At 37°C, deletion of *agrA* resulted in a significantly faster growth under all conditions tested. When NaCl was added to TSB, results differed. Indeed at 25°C, the growth of *L. monocytogenes* DG125A was affected in TSB supplemented with 1% (w/v), 3% (w/v) and 5% (w/v) NaCl. At 37°C, after addition of NaCl, the differences were significant when 3% and 5% NaCl were added to TSB.

**Table 2:** Growth rates (h^−1^) of *L. monocytogenes* EGD-e and *L. monocytogenes* DG125A during incubation at 25°C and 37°C in complex and defined medium.

Medium^a^	25°C		37°C	
	EGD-e	DG125A	EGD-e	DG125A
TSB	0.315±0.025	0.296±0.031	0.476±0.032*	0.522±0.024*
TSB 1% NaCl	0.266±0.007*	0.251±0.006*	0.451±0.019	0.477±0.009
TSB 3% NaCl	0.203±0.006*	0.179±0.003*	0.272±0.029*	0.162±0.030*
TSB 5% NaCl	0.121±0.001*	0.079±0.004*	0.244±0.013*	0.211±0.006*
KRM 0.1% casamino	0.138±0.003*	0.159±0.002*	0.072±0.002*	0.082±0.002*
KRM 1%casamino	0.137±0.004*	0.149±0.003*	0.090±0.003*	0.103±0.003*

* significant differences (P<0.05).

a NaCl and casamino acids were added on a weight per volume basis.

### Inactivation of AgrA Enhances Biofilm Formation at 37**°**C

**Figure 7:**
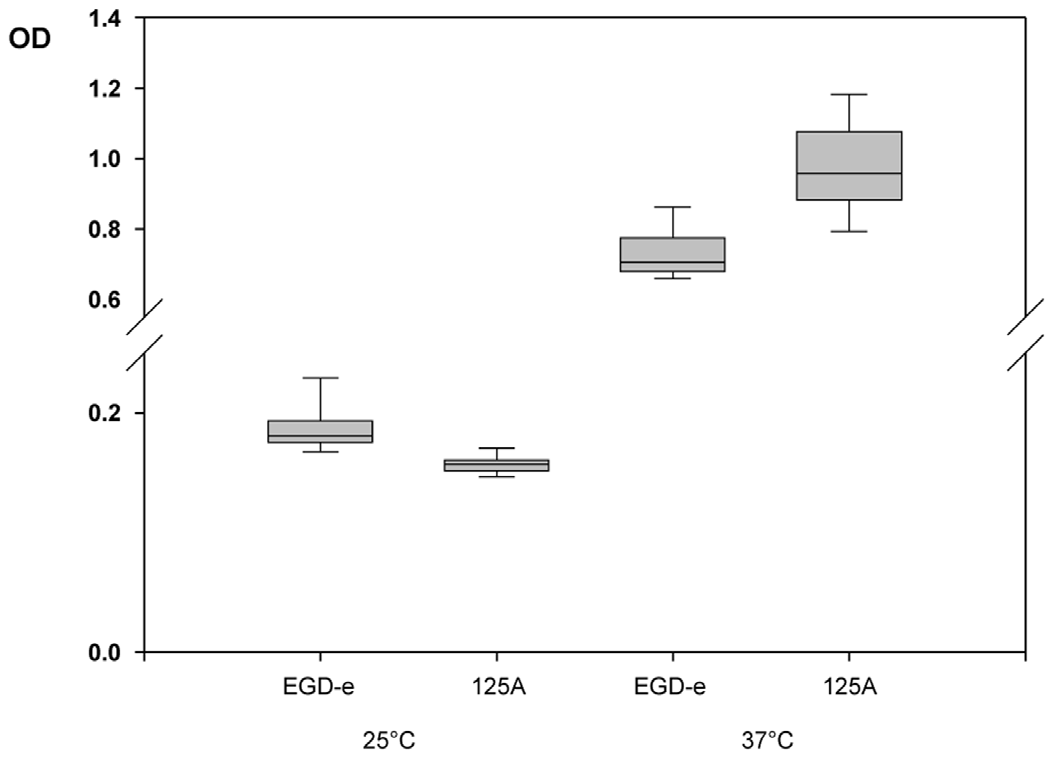
Biofilm formation after 16 h of incubation at 25°C and 37°C. The cells in biofilms were stained with cristal violet and measured using OD590. The values are medians of three independent experiments including 5 replicates per experiment. Bars represent standard deviations. The box limits represent the 5th and 95th percentiles.

Under our experimental conditions, deletion of *agrA* increased the rate of growth at 37°C. We then assessed whether this growth advantage could be observed under sessile conditions. Indeed, as shown figure 7, at 37°C *L. monocytogenes* DG125A produced significantly higher amounts of biofilm than *L. monocytogenes* EGD-e. As described before [15] deletion of *agrA* resulted in a reduction of the ability of *L. monocytogenes* to form biofilms at 25°C. Finally, both strains produced higher amounts of biofilm at 37°C than at 25°C.

## Discussion

Temperature is an important environmental cue that participates to the transition of *Listeria monocytogenes* from saprophytic life to *in vivo* adaptation and infection. Consistent with previous functional genomics studies [18], [38], [39], [40], under our experimental conditions, temperature-dependent transcriptional reshaping was observed. Transport systems, metabolism, ribosomal proteins, regulators, phage-related functions, motility and chemotaxis was enhanced at the saprophytic temperature while at 37°C, a limited number of genes were up-regulated including virulence genes.

One key finding extracted from the transcriptomic data is the temperature-dependent effect of the inactivation of *agrA* on transcription. Indeed, the number of genes with significant differences of transcript levels was higher at 37°C than at 25°C. This suggests that the regulatory role of AgrA is critical at the *in vivo* temperature of 37°C. Furthermore, as transcription of *agrA* was significantly higher at 37°C than at 25°C, temperature may be an important environmental cue for the regulation of the expression of AgrA. Temperature-dependent expression of the regulators PrfA [19] and MogR [21] through RNA and protein thermosensors respectively has been documented.

Our data suggest that at 37°C AgrA participates to the negative control of the transport of nitrogenous compounds (amino acids, peptides, glutamine, ammonium, spermidine/putrescine), carbohydrates, xanthine and uracil. Similarly, AgrA may be involved in the regulation of amino acids, purine and pyrimidine biosynthetic pathways. Indeed, glutamine is an optimal nitrogen source [41]. Higher transcription of the genes encoding glutamine transport proteins and the glutamate synthase was observed. This is the enzyme that catalyses conversion of glutamine to glutamate. Glutamate is the major donor of nitrogen to amino acids and nucleotides [41]. Higher transcription of the *pyr* operon encoding an uracil permease and the enzymes needed for the synthesis of uridine-monophosphate (UMP) from glutamine also suggests higher synthesis of pyrimidines and purines. Deregulation of the transcription of a large number of proteins of unknown functions was also recorded.

Higher transcription of ammonium, amino acids and oligopeptide transporters suggests that nitrogen uptake is enhanced in the *ΔagrA* background. This was confirmed experimentally as significantly faster growth was observed in defined medium supplemented with amino acids when AgrA was not functional. These deregulations resulted in a growth advantage of the *Δagr* mutant, especially during incubation of planktonic and biofilm cultures at 37°C, but at the saprophytic temperature of 25°C, deletion of *agrA* was detrimental to the growth of planktonic and biofilm cultures in TSB and salt tolerance was affected at both temperatures.

Two lines of evidence further suggest that AgrA may participate to the transition from saprophytism to *in vivo* lifestyles. First of all, transcription of mobility genes and phage-related functions was deregulated at 37°C in the *Δagr* background while these genes are known to be down-regulated at this temperature [18], [40]. Conversely, at 37°C, transcription of genes encoding the virulence factors InlA, InlB and the regulator PrfA was lower in the mutant background and virulence of *Δagr* mutants is affected in the murine model [13], [16].

As could be expected, our data support the scenario that the Agr system acts in a complex transcriptional regulatory network with interconnections between regulons. Indeed, 12 regulators were deregulated to some extent and most of them (10) varied specifically at 37°C.

Our data confirm the complex interconnections evidenced in the regulatory network of *L. monocytogenes*. Indeed, the alternative sigma factor σB regulates transcription of *mcsA*, *mscB* the modulators of the *ctsR* operon, *hrcA* and *prfA* [42], [43], [44] while σL regulates transcription of the *sigC* operon [42].

Complex overlaps among σB, σC, σH, σL, PrfA, CtsR and HrcA has been evidenced previously [42], [45], [46], [47]. Observation of overlaps with σB, σH, CodY and PrfA regulons is consistent with regulatory redundancies and synergistic control of transcription of particular sets of genes. Our data suggest a synergistic activity of the Agr system with PrfA, σB and σH regulation, and an antagonistic activity with CodY regulation at the *in vivo* temperature. This suggests the existence of an interconnected network that regulates transcription as the first stage of the fine-tuning of gene expression in the process of adaptation to ever changing environmental conditions. Our results suggest that AgrA participates to this complex regulatory network, especially in connexion with PrfA, σB, σH and CodY. In this study, the data presented reflects mid-log growth and one can speculate that expression profiles may change according to the phase of growth and/or environmental conditions. Indeed, AgrA is part of the auto-inducer peptide responsive Agr auto-induction system. As described in *Staphylococcus aureus* [48], auto-inducer peptides in the surroundings of the cell interact with the sensor AgrC, a histidine kinase that will phosphorylate AgrA. Under this scheme, biotic information is integrated in the fine-tuning of the physiological traits of the cell. It is tempting to speculate that during infection, through AgrA regulation, auto-induction mediates information on the listerial biotic status which, in turn, may modulate expression of key factors, including virulence factors, for optimizing *in vivo* adaptation and intracellular survival. Within the gut environment, this listerial biotic status may combine diffusion sensing [49], as a mean to assess proximity to epithelium, and assessment of the number of listerial cell in the vicinity. In this efficiency sensing scenario [50], AgrA regulation would optimize the fitness of the listerial cells by assessing the benefits of producing costly virulence factors according to the local environment.

## Materials and Methods

### Strains and Growth Condition

*Listeria monocytogenes* EGD-e [51] and *Listeria monocytogenes* DG125A [15] were kept frozen on glycerol at −70°C. Inocula were prepared by two successive cultures in Tryptone Soy broth (TSB; Biokar Diagnostics, Pantin, France) inoculated at 1% (vol/vol) and incubated 16h at 25°C. For transcriptomicFor transcriptomic analyses, cultures were grown in TSB to an optical density at 600 nm (OD600) of 0.4 TSB, Brain Heart Infusion (BHI; Biokar Diagnostics, Pantin, France) and the chemically defined medium KRM (Sigma) supplemented with either 0.1% or 1% casamino acids were used for planktonic growth kinetics and biofilm formation. Experiments were carried out at 25°C and 37°C under static conditions.

### RNA Isolation and cDNA Synthesis

As mentioned above, TSB cultures were grown to an OD600 of 0.4. In order to stabilize RNA before extraction, cultures were immediately treated with RNAprotect reagent (QIAGEN, Courtaboeuf, France) according to the manufacturer’s instructions. For each experimental condition, three independent biological replicates were prepared and treated separately.

Stabilised cultures were centrifuged (5500 g, 5 min) and the bacterial pellet was suspended in 700 µl RLT buffer (Qiagen RNeasy kit) supplemented with 1% β-mercaptoethanol; 0.2 g of RNAse-free glass beads (100 µm) was added and cells were disrupted mechanically in a Fast Prep (MP Bio, France) with 4 cycles (6 m/s; 30 s).

Total RNA was isolated using Qiagen RNeasy kit according to the manufacturer’s protocol. A first on-column DNase I (10 U, Quiagen) treatment was performed prior to elution. Subsequently, 20 µg RNA were treated with 25U RQ1 RNase-free DNase I (Promega, Charbonnière, France) in the presence of RNasin (40 U, Promega) and RNA was purified with Qiagen RNeasy MinElute cleanup kit according to the manufacturer’s instructions. RNA purity and quality were assessed spectrophotometrically at 260 nm and by gel electrophoresis under denaturing conditions. 10 µg of RNA were reverse transcribed using Superscript Double-Stranded cDNA Synthesis kit (Invitrogen, Cergy Pontoise, France) according to the manufacturer’s instructions.

### Whole-genome Microarrays and Data Analysis

A custom whole-genome microarray including all the 2857 annotated open reading frames (ORFs) of the genome of *Listeria monocytogenes* EGD-e was designed by NimbleGen Systems. Microarray hybridization, washes, raw data pre-processing and normalization was performed by NimbleGen Systems Inc (Madison, WI) according to their standard protocol.

DNASTAR ArrayStar software (Madison, WI) was used for the analysis of normalized results. From the three independent datasets, T statistics and *P* values (*P*<0.05) were calculated to determine differentially expressed genes.

Several differential analyses were performed. First of all, for each strain, results were compared on the basis of the growth temperature (25°C versus 37°C). The fold change was calculated by comparing results at 25°C with expression levels at 37°C. Genes with at least 2-fold change were considered for interpretation in order to identify the relative importance of specific processes based on categories according to the functional classification established for the genus *Listeria* [12]. Gene ontology was subsequently searched to identify relationships between genes with particular biological functions. Z-Scores, the standard statistical test used for hypergeometric distributions and the hypergeometric probability distribution (P-value) were calculated in order to determine the chance that a certain number of genes will be selected for any given gene ontology term.

In a second step, results were analysed to identify set of genes with significant differences according to the genetic background. The fold change was calculated by comparing results of strain DG125A with expression levels of strain EGD-e. This analysis was done on the results collected at 25°C and 37°C.

Finally, results were processed to identify members of the σB [52], σC [45], σL [45], σH [45], CodY [28], CtsR [46], HrcA [42], PrfA [47] and VirR [53] regulons in the sets of genes identified as significant. “σB”, “σC”, “σL”, “σH”, “CodY”, “CtsR”, “HrcA”, “PrfA”, “VirR”, “co-regulation by PrfA and σB” were included in the gene ontology analysis.

For each condition, three sets of cDNAs were prepared from three sets of RNAs extracted from three independent biological repetitions and hybridised independently. Microarray data have been deposited to the Gene Expression Omnibus (https://www.ncbi.nlm.nih.gov/geo/).

### Real Time PCR

In order to confirm results of the microarray analysis, transcript levels of five genes with higher and lower transcript levels were quantified using SYBR Green real time qRT-PCR as described previously [15]. Primers were designed using Primer3 (http://frodo.wi.mit.edu/primer3/) and tested with genomic DNA prior to analysis. Three sets of cDNA originating from three sets of RNA extracted after growth at 25°C and 37°C were analysed. The double ΔCt method was used to analyse qRT-PCR results. The housekeeping gene *ldh* was selected as reference.

### Planktonic Growth

Three hundred microlitres of inoculated cultures were dispensed into wells of a Bioscreen™ microtiter plates. Five wells were inoculated per culture. OD_600_ readings were monitored every 15 minutes with a Bioscreen™ and growth was followed for 24 h. Data representing growth of three independent cultures were collected. The growth rate of each independent growth curve was calculated. The significance of differences between strains was determined by analysis of variance (P<0.05) with SigmaStat™.

### Biofilm Formation

The microtiter plate assay [54] was used for quantification of biofilms. Briefly, cultures were grown on polystyrene microplates (Nunc; Dominique Dutscher S.A., Brumath, France). Inocula were used to inoculate (1/100, vol/vol) fresh medium and the culture was grown to an OD600 of 0.05. One hundred microliters of the culture were transferred into a microtiter plate well, and the plate was tightly sealed and incubated for 16 h. After incubation, the medium was removed from each well, and the plates were washed twice with saline solution (150mM) using a microtiter plate washer (Cellwash; Thermolabsystems, Cergy Pontoise, France) in order to remove loosely attached cells. Plates were stained 45 min with an aqueous crystal violet (0.05% (wt/vol)). After four washes, 100 µl of an alcoholic destaining solution (96% vol/vol) was added to each well, and the optical density at 595 nm was determined. For each experiment, 15 replicates resulting from 3 different inocula were analyzed. The variance of the results was analyses to determine the significance of the differences (P<0.05).

## Supporting Information

Supplementary Material S1 **Complementation of** ***L. monocytogenes*** **DG125A with the parental version of** ***agrA*** **and results of the comparison of transcripts of** ***agrA*** **,** ***lmo0477*** **,** ***prfA*** **and lmo** ***1972*** **in the complemented mutant and parental strains.**
(PDF)

Table S1 **List of genes with higher transcripts levels specifically in** ***L. monocytogenes*** **EGD-e at 25°C.**
(PDF)

Table S2 **List of genes with higher transcripts levels specifically in** ***L. monocytogenes*** **DG125A at 25°C.**
(PDF)

Table S3 **List of genes with higher transcripts levels specifically in** ***L. monocytogenes*** **EGD-e at 37°C.**
(PDF)

Table S4 **List of genes with higher transcripts levels specifically in** ***L. monocytogenes*** **DG125A at 37°C.**
(PDF)

Table S5 **List of genes with transcripts variations in the analysis DG125A versus EGD-e specific to 25°C.**
(PDF)

Table S6 **List of genes with transcripts variations in the analysis DG125A versus EGD-e specific to 37°C.**
(PDF)

Table S7 **List of genes with similar trends at 25°C and 37°C in the analysis DG125A versus EGD-e.**
(PDF)
