# Supplementary material for: Listeria monocytogenes Differential Transcriptome Analysis Reveals Temperature-Dependent Agr Regulation and Suggests Overlaps with Other Regulons
Source: PLoS One. 2012 Sep 14;7(9):e43154. doi: 10.1371/journal.pone.0043154 (PMC3443086; doi:10.1371/journal.pone.0043154)
Supplement: Supplementary Material S1 — Complementation of L. monocytogenes DG125A with the parental version of agrA and results of the comparison of transcripts of agrA , lmo0477 , prfA and lmo 1972 in the complemented mutant and parental strains. (PDF) [file pone.0043154.s001.pdf]

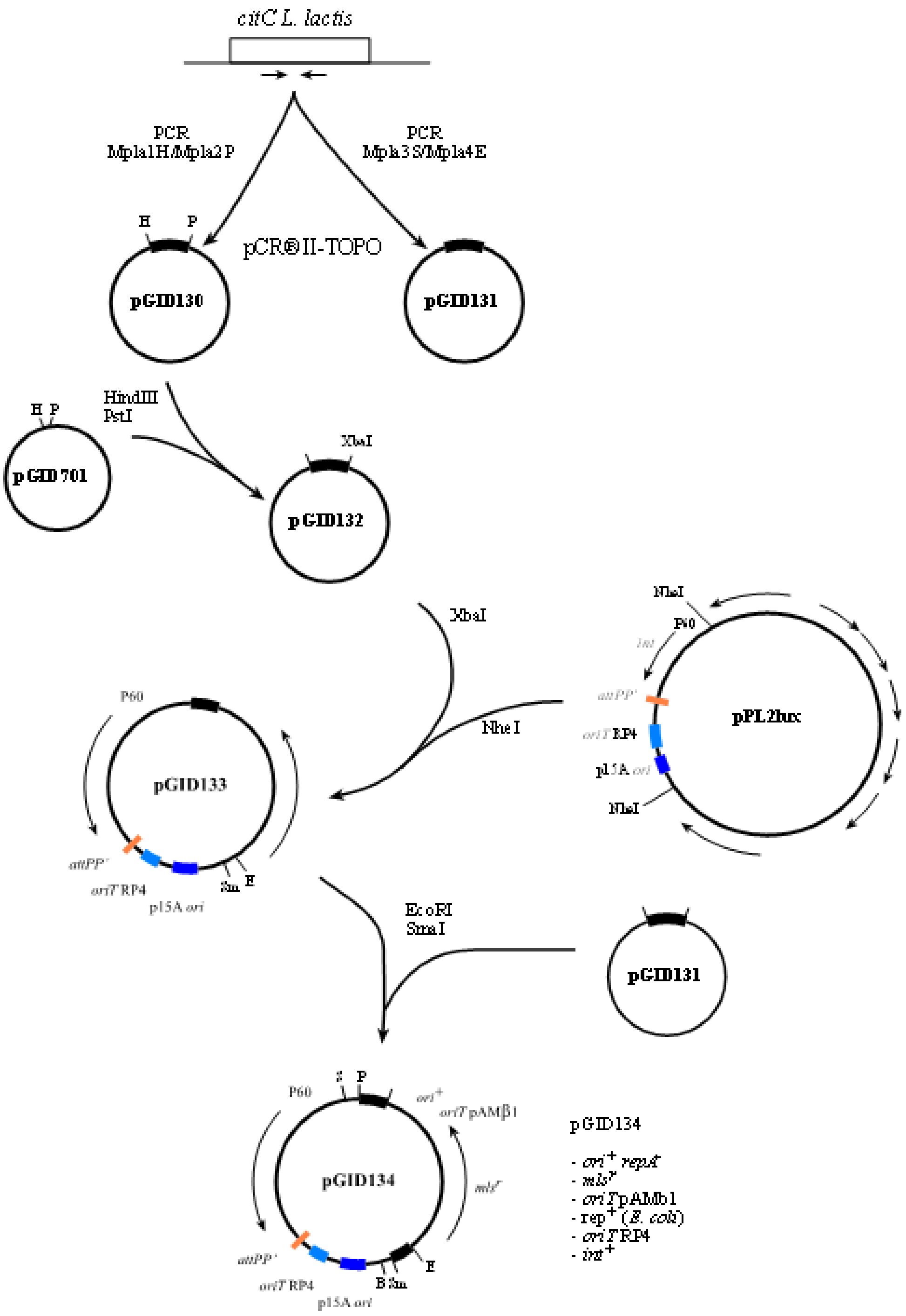
citC L. lactis
PCR
Mpla1H/Mpla2P
PCR
Mpla3S/Mpla4E
H P
pCR®II-TOPO
pGID130
pGID131
HindIII
PstI
H P
pGID701
XbaI
pGID132
XbaI
NheI
P60
int
attPP'
oriT RP4
p15A ori
NheI
pPL2lux
NheI
P60
pGID133
attPP'
oriT RP4
p15A ori
Sm E
EcoRI
SmaI
pGID131
S P
P60
ori+
oriT pAMβ1
pGID134
mls^r
attPP'
oriT RP4
p15A ori
B Sm
E
pGID134
- ori+ repA
- mls^r
- oriT pAMb1
- rep+ (E. coli)
- oriT RP4
- int+

Figure S1.1

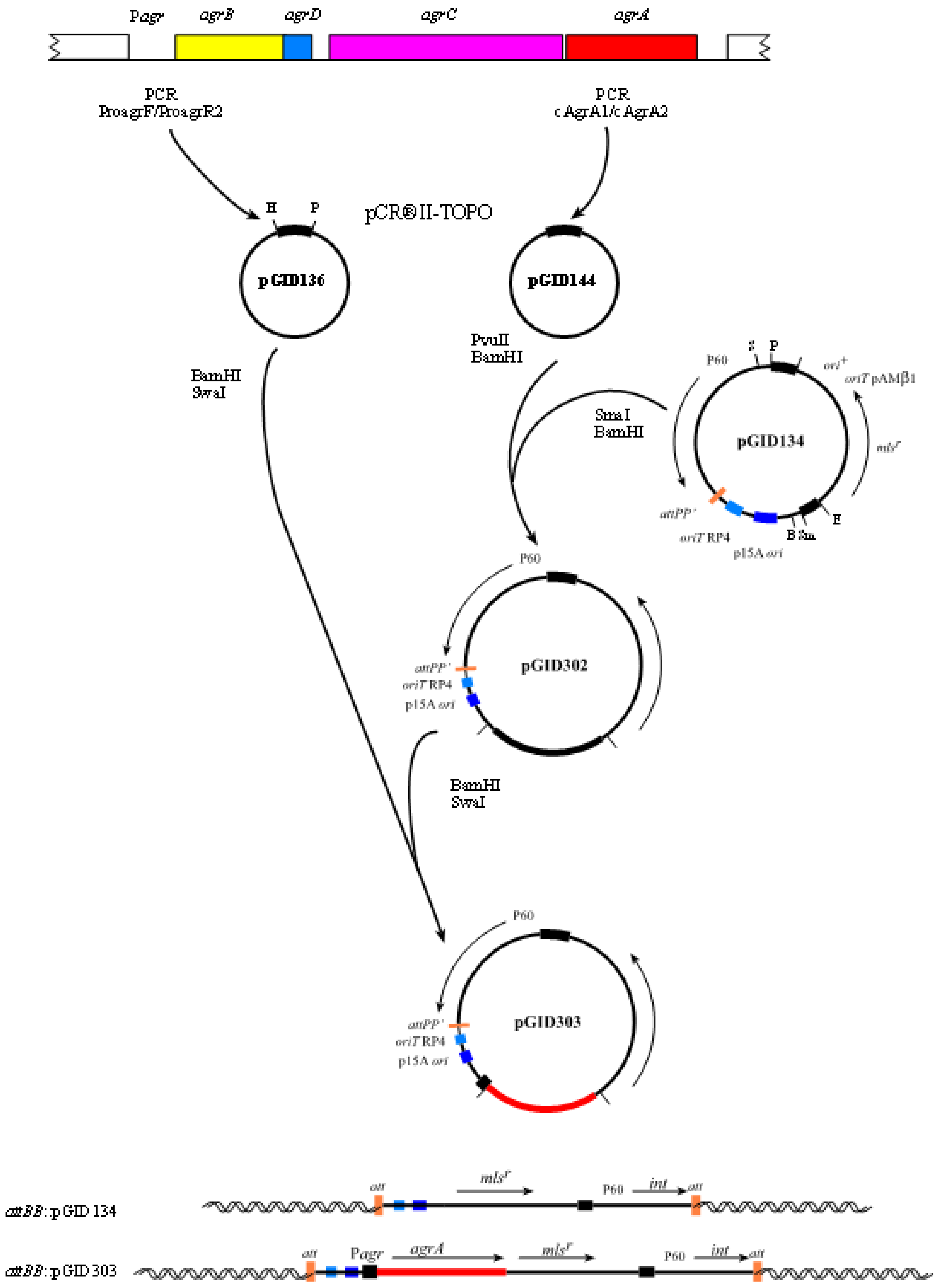

Figure S1.1 continued

Figure S1.1. Construction of plasmid and *Listeria monocytogenes* strains.

The internal fragment of *citC* was PCR amplified (with Mpla1H/Mpla2P or Mpla3S/Mpla4E) from *Lactococcus lactis* and cloned into pCR®II-TOPO (Invitrogen). The HindIII/PstI fragment was transferred into the pGID701 (ref), yielding pGID132. A NheI fragment from pPL2lux (ref) was cloned into XbaI restriction site. The resultant plasmid was designated pGID133. A second citC fragment from pGID131 was introduced into pGID133, generating pGID134.

The promoters Pagr and CDS of *agrA* were PCR amplified from *L. monocytogenes* EGD-e chromosomal DNA using the primer couples ProagrF/ProagrR2 and cAgrA1/cAgrA2 respectively. The resultant plasmids were designated pGID136 and pGID144.

A BamHI/PvuII fragment harbouring completed CDS of *agrA* was cloned into pGID134 cut with SmaI and BamHI. The Pagr promoter (fragment BamHI/SwaI) was cloned into the BamHI/SwaI restriction site of pGID302, generating pGID303.

The plasmid pGID134 and pGID303 were electroporated into *L. monocytogenes* EGD-e and DG125A and integrated into the PSA attachment site ($tRNA_{Arg}$-*attBB*).

The P*agr-agrA* was verified by sequencing.

Table S1.1. Plasmids, Strains and Oligonucleotides

Name	Plasmid<br>Description	Reference
pCR®II-TOPO	Cloning vector from Invitrogen	
pGID701	Em^r, ori$_+$ of pWV01, replicates only in strains providing repA in trans	1
pPL2Lux	Site-specific listerial integrative vector, Cm^r	3
pGID134	Site-specific listerial integrative vector, Em^r	This study
pGID303	Site-specific listerial integrative vector, P*agr-agrA*,Em^r	This study
	Bacteria	
EGD-e	Parental serotype 1/2a strain, genome sequenced	2
DG125A		4
DG140E	EGD-e transformed with pGID134 integrated at the tRNA$_{ARG}$.	This study
DG164E	EGD-e transformed with pGID303 integrated at the tRNA$_{ARG}$.	This study
DG165A	EGD-e transformed with pGID303 integrated at the tRNA$_{ARG}$.	This study
	Oligonucleotide	
Name		RE site
Mpla1H	aa**aagctt**gacttgcactaagggcgaac	HindIII
Mpla2P	aa**ctgcag**cccagcgtaaatggattagc	PstI
Mpla3S	aa**cccggg**gacttgcactaagggcgaac	SmaI
Mpla4E	cccagcgtaaatggattagc	
ProagrF	gtgaacattctcgagcagttggtt	
ProagrR2	**atttaaat**tttacaacgaatttacccata	SwaI
cAgrA1	gt**atttaaat**ggggatgaatttatgcta	SwaI
cAgrA2	aaa**ctgcag**caaatccaaacttgccattgt	Pst1

Restriction sites are in bold type

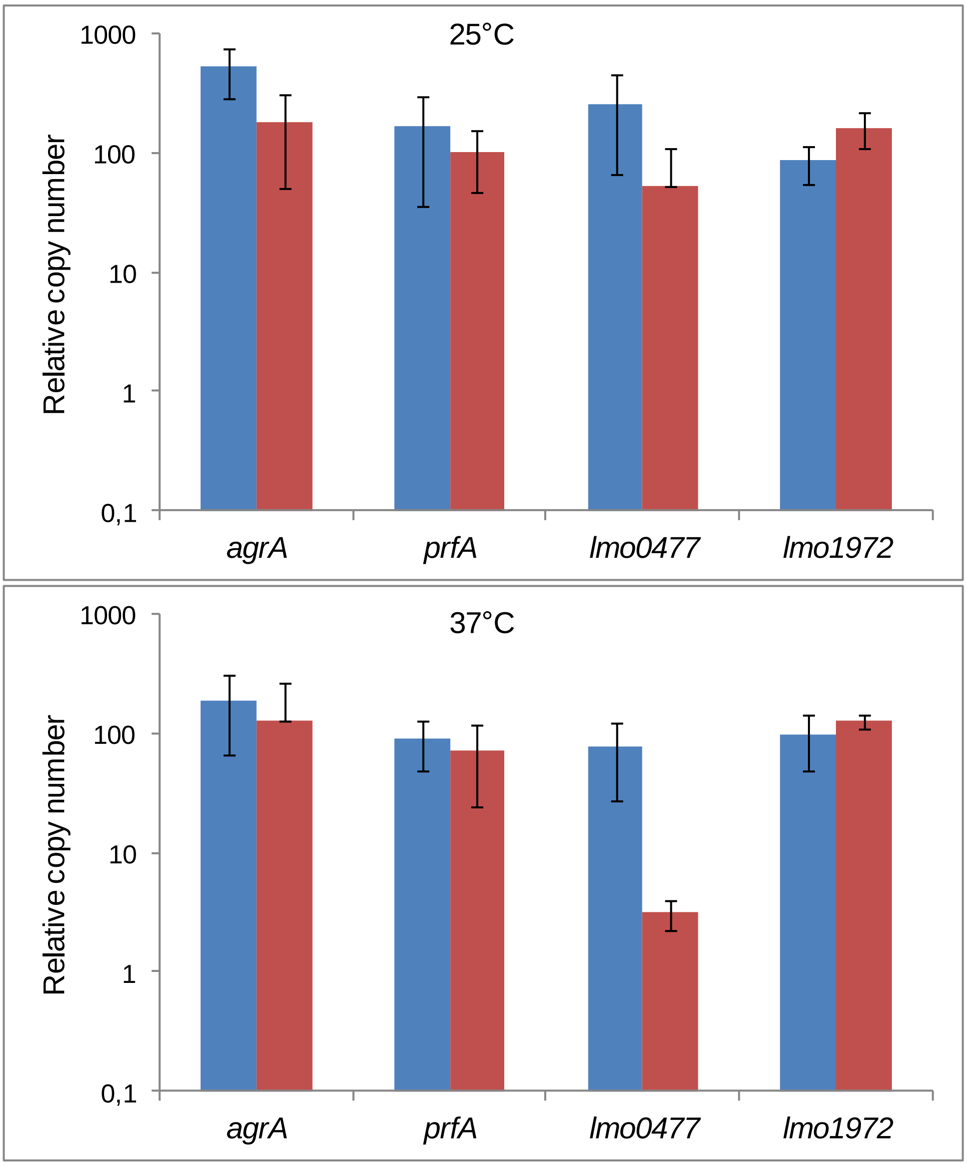

Figure S1.2. Comparison of the relative copy numbers of transcripts of *Listeria monocytogenes* DG164E (blue) and DG165A (red).

Transcripts were quantified by reverse transcription real time PCR. Copy numbers of targets per PCR well were determined according to Rutledge and Stewart (5-6) by the "Linear Regression of Efficiency" (LRE) method for a more accurate determination of PCR efficiency. Based on the ability to determine target quantity directly from fluorescence readings within the central region of an amplification profile, the LRE Analyzer allows absolute quantification to be conducted with little or no user intervention. Amplification efficiency and the regression coefficient R^2 for each sample were calculated from the fluorescence raw data with the LRE analyzer software (sites.google.com/site/lreqpcr) (7). Results are presented after standardisation with the copy numbers of *L. monocytogenes* DG140E. ANOVA indicated that the differences of copy number between the two strains were not significant.

References

1. **Beltramo, C., M. Oraby, G.Bourel, D. Garmyn, and J. Guzzo.** 2004. A new vector, pGID052, for genetic transfer in *Oenococcus oeni*. FEMS Microbiology Letters **236**: 53–60

2. **Murray, E. G. D., R. A. Webb, and M. B. R. Swann.** 1926. A disease of rabbit characterised by a large mononuclear leucocytosis, caused by a hitherto undescribed bacillus: *Bacterium monocytogenes* (n. sp.). J. Pathol. Bacteriol. **29:**407–439

3. **Riedel, C. U., I. R. Monk, P. G. Casey, D. Morrissey, G. C. O'Sullivan, M. Tangney, C. Hill, and C. G. Gahan**. 2007. Improved luciferase tagging system for *Listeria monocytogenes* allows real-time monitoring in vivo and in vitro. Appl. Environ. Microbiol. **73:**3091–3094.

4. **Rieu, A., S. Weidmann, D. Garmyn, P. Piveteau, and J. Guzzo.** 2007. *agr* system of *Listeria monocytogenes* EGD-e: role in adherence and differential expression pattern. Appl. Environ. Microbiol. **73:**6125–6133.

5. **Rutledge RG, and D**. **Stewart.** 2008. A kinetic-based sigmoidal model for the polymerase chain reaction and its application to high-capacity absolute quantitative real-time PCR. BMC Biotechnol **8**: 47

6. **Rutledge RG, and D. Stewart.** 2008. Critical evaluation of methods used to determine amplification efficiency refutes the exponential character of real-time PCR. BMC Mol Biol **9**: 96.

7. **Rutledge RG, and D**. **Stewart.** 2010. Assessing the performance capabilities of LRE-based assays for absolute quantitative real-time PCR. PLoS ONE **5**: e9731.

8. **Rutledge RG, and D**. **Stewart.** 2011. A Java program for LRE-based real-time qPCR that enables large-scale absolute quantification. PLoS ONE **6**: e17636
