# Supplementary material for: Listeria monocytogenes Differential Transcriptome Analysis Reveals Temperature-Dependent Agr Regulation and Suggests Overlaps with Other Regulons
Source: PLoS One. 2012 Sep 14;7(9):e43154. doi: 10.1371/journal.pone.0043154 (PMC3443086; doi:10.1371/journal.pone.0043154)
Supplement: Table S1 — List of genes with higher transcripts levels specifically in L. monocytogenes EGD-e at 25°C. (PDF) [file pone.0043154.s002.pdf]

name	Functional category	EGD-e 25°C versus 37°C	125A 25°C versus 37°C
RsbR	4.1	2,425 up	1,813 up
adeC	2.3	3,092 up	1,677 up
ansB		3,147 up	1,943 up
argD	2.2	3,981 up	1,867 up
argS	3.7.2	2,046 up	1,140 up
aroB	2.2	2,817 up	1,490 up
atpA	1.4	2,128 up	1,643 up
atpH	1.4	2,418 up	1,889 up
bvrC	5.2	2,273 up	1,868 up
cadA	1.2	2,402 up	1,343 up
cdsA	2.4	2,144 up	1,069 down
cinA	1.10	2,989 up	1,948 up
citZ	2.1.3	2,499 up	1,093 up
cobD	2.5	2,494 up	1,770 up
comEC	1.10	2,436 up	1,490 up
cydC	1.2	2,394 up	1,966 up
cysS	3.7.2	2,586 up	1,818 up
dapB	2.2	3,161 up	1,895 up
dltB	1.1	2,827 up	1,427 up
dnaA	3.1	2,089 up	1,588 up
dnaB	3.1	3,189 up	1,844 up
dnaG	3.1	2,047 up	1,292 up
drm	2.3	3,171 up	1,895 up
folD	2.5	2,672 up	1,436 up
ftsA	1.7	2,302 up	1,533 up
ftsH	1.7	2,350 up	1,578 up
gatA	3.7.2	3,328 up	1,857 up
gbuA	1.2	2,118 up	1,092 up
glyQ	3.7.2	2,694 up	1,384 up
glyS	3.7.2	3,527 up	1,995 up
guaB	2.3	2,096 up	1,559 up
gyrB	3.4	2,092 up	1,787 up
hemA	2.5	2,383 up	1,543 up
hemB	2.5	2,711 up	1,374 up
hemC	2.5	2,217 up	1,235 up
hemL	2.5	2,224 up	1,694 up
hisA	2.2	2,772 up	1,923 up
hisB	2.2	3,311 up	1,779 up
hisF	2.2	3,185 up	1,971 up
hisG	2.2	2,118 up	1,362 up
hisS	3.7.2	2,871 up	1,634 up
hom	2.2	2,545 up	1,901 up
hrcA	3.5.2	2,121 up	1,695 up
ileS	3.7.2	2,227 up	1,232 up
ilvD	2.2	2,194 up	1,360 up
ilvN	2.2	2,592 up	1,571 up
infB	3.7.3	2,959 up	1,685 up

name	Functional category	EGD-e 25°C versus 37°C	125A 25°C versus 37°C
leuB	2.2	2,790 up	1,609 up
leuC	2.2	2,718 up	1,188 up
leuS	3.7.2	2,886 up	1,541 up
lisK	1.3	2,669 up	1,644 up
lisR	3.5.2	2,810 up	1,726 up
lmo0020	3.5.2	2,192 up	1,559 up
lmo0041	3.5.2	2,361 up	1,667 up
lmo0061	5.2	3,266 up	1,479 up
lmo0064	6.0	2,466 up	1,861 up
lmo0071	6.0	2,246 up	1,034 down
lmo0072	6.0	2,163 up	1,068 up
lmo0073	5.1	3,533 up	1,854 up
lmo0074	5.1	3,601 up	1,431 up
lmo0075	2.1.1	2,500 up	1,377 up
lmo0135	1.2	2,460 up	1,478 up
lmo0140	5.1	2,877 up	1,953 up
lmo0143	6.0	2,945 up	1,739 up
lmo0178	3.5.2	2,638 up	1,292 up
lmo0179	1.2	4,060 up	1,860 up
lmo0181	1.2	3,508 up	1,822 up
lmo0183	2.1.1	2,654 up	1,467 up
lmo0240	5.2	2,059 up	1,639 up
lmo0267	5.2	2,361 up	1,856 up
lmo0271	2.1.1	2,122 up	1,446 up
lmo0276	5.2	2,304 up	1,844 up
lmo0290	5.2	2,389 up	1,899 up
lmo0304	5.2	3,019 up	1,516 up
lmo0312	5.2	2,346 up	1,016 up
lmo0380	6.0	2,168 up	1,939 up
lmo0390	3.2	2,242 up	1,863 up
lmo0391	6.0	2,797 up	1,869 up
lmo0455	5.2	2,039 up	1,764 up
lmo0458	2.2	2,081 up	1,241 up
lmo0460	1.8	2,043 up	1,532 up
lmo0503	1.2	2,161 up	1,860 up
lmo0506	2.1.1	2,485 up	1,179 up
lmo0530	5.2	2,723 up	1,313 up
lmo0533	5.2	2,887 up	1,778 up
lmo0542	1.2	2,438 up	1,870 up
lmo0571	3.2	2,496 up	1,975 up
lmo0586	1.8	2,021 up	1,712 up
lmo0598	2.5	3,706 up	1,369 up
lmo0618	3.8	2,691 up	1,862 up
lmo0636	5.2	2,126 up	1,987 up
lmo0664	2.1.1	3,023 up	1,970 up
lmo0666	5.2	2,044 up	1,952 up
lmo0756	1.2	2,976 up	1,962 up

name	Functional category	EGD-e 25°C versus 37°C	125A 25°C versus 37°C
lmo0757	5.2	2,571 up	1,553 up
lmo0765	5.2	5,743 up	1,246 up
lmo0767	1.2	5,605 up	1,123 down
lmo0768	1.2	4,589 up	1,784 up
lmo0769	2.1.1	2,717 up	1,041 down
lmo0774	5.2	2,360 up	1,728 up
lmo0776	3.5.2	2,095 up	1,454 up
lmo0787	1.2	2,702 up	1,317 up
lmo0802	2.3	2,114 up	1,506 up
lmo0806	3.5.2	2,852 up	1,624 up
lmo0807	1.2	5,277 up	1,852 up
lmo0808	1.2	3,042 up	1,176 down
lmo0809	1.2	2,312 up	1,178 down
lmo0810	1.2	2,335 up	1,032 up
lmo0823	2.1.1	2,874 up	1,893 up
lmo0826	1.2	2,151 up	1,163 up
lmo0841	1.2	2,408 up	1,657 up
lmo0847	1.2	3,031 up	1,169 down
lmo0859	1.2	2,050 up	1,688 up
lmo0862	2.1.1	2,883 up	1,610 up
lmo0883	5.2	2,117 up	1,481 up
lmo0887	5.2	2,413 up	1,815 up
lmo0888	5.2	2,903 up	1,661 up
lmo0901	1.2	2,719 up	1,558 up
lmo0906	4.1	2,136 up	1,698 up
lmo0936	1.4	2,106 up	1,441 up
lmo0940	5.2	2,321 up	1,348 up
lmo0951	5.2	2,460 up	1,480 up
lmo0960	2.2	2,157 up	1,970 up
lmo0961	2.2	2,034 up	1,707 up
lmo0966	5.2	2,933 up	1,874 up
lmo0969	3.6	3,023 up	1,773 up
lmo0983	4.1	2,230 up	1,630 up
lmo0985	6.0	2,057 up	1,084 up
lmo0986	1.2	2,210 up	1,194 up
lmo0987	5.2	2,915 up	1,587 up
lmo0991	5.2	3,167 up	1,248 up
lmo0992	5.2	2,188 up	1,044 up
lmo1005	2.4	2,486 up	1,670 up
lmo1011	2.2	2,065 up	1,530 up
lmo1019	5.2	2,633 up	1,701 up
lmo1020	5.2	2,064 up	1,152 up
lmo1021	1.3	2,286 up	1,575 up
lmo1022	3.5.2	2,420 up	1,724 up
lmo1025	5.2	2,434 up	1,239 up
lmo1031	5.2	5,003 up	1,891 up
lmo1039	1.2	3,018 up	1,769 up

name	Functional category	EGD-e 25°C versus 37°C	125A 25°C versus 37°C
lmo1046	2.5	3,578 up	1,642 up
lmo1073	1.2	2,104 up	1,133 down
lmo1076	1.1	2,631 up	1,965 up
lmo1081	1.1	2,125 up	1,533 up
lmo1082	1.1	2,792 up	1,530 up
lmo1083	1.1	2,296 up	1,264 up
lmo1085	1.1	2,577 up	1,407 up
lmo1091	1.1	4,561 up	1,795 up
lmo1092	5.2	2,995 up	1,519 up
lmo1095	1.2	2,357 up	1,821 up
lmo1097	4.4	2,380 up	1,342 up
lmo1101	1.6	2,342 up	1,634 up
lmo1104	1.1	2,270 up	1,997 up
lmo1105	4.4	2,103 up	1,556 up
lmo1129	5.2	3,034 up	1,620 up
lmo1135	6.0	2,389 up	1,481 up
lmo1153	2.1	2,827 up	1,576 up
lmo1154	2.1.1	3,016 up	1,997 up
lmo1156	2.1.1	2,985 up	1,829 up
lmo1157	2.1.1	2,005 up	1,281 up
lmo1158	2.1.1	2,078 up	1,737 up
lmo1159	2.1.1	2,194 up	1,845 up
lmo1161	2.1.1	2,273 up	1,205 up
lmo1164	2.1.1	2,367 up	1,552 up
lmo1165	2.1.1	2,454 up	1,476 up
lmo1170	5.2	2,273 up	1,668 up
lmo1181	2.5	2,581 up	1,423 up
lmo1185	2.1.1	2,227 up	1,958 up
lmo1218	3.6	2,469 up	1,949 up
lmo1223	1.2	3,131 up	1,306 up
lmo1224	5.2	3,060 up	1,422 up
lmo1225	3.5.2	2,195 up	1,352 up
lmo1226	1.2	2,129 up	1,130 down
lmo1232	3.2	2,661 up	1,187 up
lmo1243	5.2	2,293 up	1,924 up
lmo1269	1.6	2,182 up	1,253 up
lmo1307	6.0	2,350 up	1,210 down
lmo1311	5.2	2,784 up	1,010 up
lmo1315	1.1	3,658 up	1,679 up
lmo1317	2.4	2,739 up	1,717 up
lmo1326	5.2	2,099 up	1,635 up
lmo1337	5.2	2,016 up	1,316 up
lmo1338	5.2	2,206 up	1,378 up
lmo1339	2.1.1	2,741 up	1,629 up
lmo1349	2.2	4,308 up	1,705 up
lmo1350	2.2	2,648 up	1,908 up
lmo1353	5.2	3,475 up	1,449 up

name	Functional category	EGD-e 25°C versus 37°C	125A 25°C versus 37°C
lmo1354	2.2	3,575 up	1,899 up
lmo1356	2.4	2,716 up	1,936 up
lmo1357	2.4	2,614 up	1,572 up
lmo1358	5.2	3,201 up	1,643 up
lmo1359	3.5.4	3,399 up	1,420 up
lmo1361	2.3	3,842 up	1,810 up
lmo1362	2.3	2,984 up	1,947 up
lmo1369	2.4	2,075 up	1,406 up
lmo1370	2.4	2,177 up	1,260 up
lmo1371	2.4	2,630 up	1,916 up
lmo1372	2.4	3,287 up	1,881 up
lmo1385	5.2	2,378 up	1,463 up
lmo1389	1.2	2,885 up	1,589 up
lmo1390	1.2	2,491 up	1,054 up
lmo1391	1.2	2,196 up	1,115 up
lmo1394	2.4	2,035 up	1,304 up
lmo1395	5.2	2,284 up	1,040 down
lmo1396	2.4	2,404 up	1,313 up
lmo1399	5.2	2,733 up	1,917 up
lmo1415	2.4	2,886 up	1,815 up
lmo1417	5.2	4,302 up	1,725 up
lmo1420	1.1	2,111 up	1,510 up
lmo1434	5.2	2,576 up	1,874 up
lmo1435	2.2	2,645 up	1,552 up
lmo1436	2.2	2,917 up	1,736 up
lmo1437	2.2	2,528 up	1,677 up
lmo1438	1.1	2,716 up	1,516 up
lmo1441	1.1	2,248 up	1,422 up
lmo1453	5.2	3,025 up	1,039 down
lmo1456	5.2	2,750 up	1,603 up
lmo1463	2.3	2,322 up	1,382 up
lmo1464	2.4	2,563 up	1,354 up
lmo1465	5.2	2,491 up	1,327 up
lmo1466	5.2	2,018 up	1,527 up
lmo1467	2.6	2,010 up	1,146 up
lmo1488	5.2	2,653 up	1,389 up
lmo1489	5.2	3,327 up	1,862 up
lmo1490	2.2	2,730 up	1,679 up
lmo1491	5.2	3,019 up	1,514 up
lmo1492	5.2	2,810 up	1,296 up
lmo1498	4.5	2,201 up	1,013 up
lmo1500	5.2	2,341 up	1,561 up
lmo1507	3.5.2	3,467 up	1,830 up
lmo1508	1.3	2,290 up	1,319 up
lmo1510	5.2	2,034 up	1,310 up
lmo1511	5.2	2,160 up	1,227 up
lmo1516	1.2	2,169 up	1,861 down

name	Functional category	EGD-e 25°C versus 37°C	125A 25°C versus 37°C
lmo1517	2.2	2,170 up	1,606 down
lmo1518	6.0	2,607 up	1,786 up
lmo1528	5.2	2,456 up	1,695 up
lmo1531	3.6	2,430 up	1,376 up
lmo1537	4.5	2,020 up	1,101 down
lmo1555	2.5	3,087 up	1,926 up
lmo1568	5.2	2,085 up	1,044 down
lmo1576	5.2	2,706 up	1,631 up
lmo1577	5.2	2,435 up	1,789 up
lmo1585	2.2	2,730 up	1,733 up
lmo1594	1.7	2,153 up	1,987 up
lmo1597	6.0	4,629 up	1,881 up
lmo1603	2.2	2,738 up	1,504 up
lmo1611	2.2	2,082 up	1,447 up
lmo1621	3.2	2,419 up	1,911 up
lmo1635	5.2	2,257 up	1,860 up
lmo1636	1.2	2,283 up	1,064 up
lmo1646	3.3	2,234 up	1,018 up
lmo1651	1.2	2,238 up	1,173 up
lmo1652	1.2	3,473 up	1,638 up
lmo1654	6.0	2,651 up	1,342 up
lmo1669	5.2	2,724 up	1,745 up
lmo1674	3.8	2,498 up	1,314 up
lmo1689	3.2	2,522 up	1,043 down
lmo1708	4.2	2,977 up	1,702 up
lmo1711	2.2	2,926 up	1,536 up
lmo1712	1.2	2,389 up	1,057 up
lmo1715	5.2	2,504 up	1,346 up
lmo1719	1.2	2,540 up	1,676 up
lmo1722	3.6	2,114 up	1,503 up
lmo1727	3.5.2	2,810 up	1,767 up
lmo1728	2.1.1	3,231 up	1,682 up
lmo1730	1.2	3,015 up	1,938 up
lmo1737	2.1.1	2,571 up	1,596 up
lmo1743	6.0	2,262 up	1,298 up
lmo1758	3.1	2,177 up	1,957 up
lmo1761	1.2	2,682 up	1,177 up
lmo1762	5.1	3,287 up	1,754 up
lmo1795	5.2	2,228 up	1,540 up
lmo1802	5.2	2,796 up	1,865 up
lmo1810	5.2	2,496 up	1,107 up
lmo1811	3.3	2,143 up	1,200 up
lmo1812	2.2	3,489 up	1,972 up
lmo1813	2.2	2,043 up	1,690 up
lmo1814	5.2	2,593 up	1,745 up
lmo1822	3.6	2,597 up	1,813 up
lmo1825	2.5	2,865 up	1,839 up

name	Functional category	EGD-e 25°C versus 37°C	125A 25°C versus 37°C
lmo1843	5.2	2,364 up	1,805 up
lmo1855	1.1	2,050 up	1,284 up
lmo1859	3.5.2	2,187 up	1,904 up
lmo1866	5.2	2,586 up	1,804 up
lmo1873	2.5	2,271 up	1,678 up
lmo1878	3.5.2	2,047 up	1,332 up
lmo1884	1.2	3,138 up	1,095 up
lmo1886	2.2	3,093 up	1,813 up
lmo1887	5.2	3,106 up	1,356 up
lmo1906	2.1.1	2,441 up	1,521 up
lmo1909	5.2	2,825 up	1,530 up
lmo1915	2.1.1	2,836 up	1,868 up
lmo1937	5.2	2,331 up	1,071 up
lmo1949	5.2	2,390 up	1,375 up
lmo1951	5.2	3,084 up	1,654 up
lmo1970	2.4	2,097 up	1,756 up
lmo1971	1.2	2,551 up	1,922 up
lmo1999	2.1.1	2,465 up	1,048 down
lmo2004	3.5.2	2,047 up	1,724 up
lmo2008	1.2	2,644 up	1,034 down
lmo2009	1.2	3,813 up	1,461 up
lmo2011	1.3	3,642 up	1,670 up
lmo2031	5.2	2,695 up	1,470 up
lmo2041	5.2	2,419 up	1,378 up
lmo2052	1.1	2,586 up	1,674 up
lmo2053	5.2	2,374 up	1,522 up
lmo2073	1.2	2,633 up	1,706 up
lmo2074	5.2	2,156 up	1,278 up
lmo2102	5.2	2,035 up	1,689 up
lmo2105	1.2	2,459 up	1,592 up
lmo2111	1.4	2,693 up	1,708 up
lmo2114	1.2	2,148 up	1,066 up
lmo2141	5.2	2,604 up	1,941 up
lmo2162	5.2	2,352 up	1,971 up
lmo2195	1.2	2,866 up	1,413 up
lmo2227	1.2	2,020 up	1,689 up
lmo2245	5.2	2,008 up	1,791 up
lmo2248	5.2	2,330 up	1,507 up
lmo2343	4.2	2,240 up	1,459 up
lmo2345	5.2	3,835 up	1,793 up
lmo2346	1.2	3,616 up	1,650 up
lmo2351	1.4	8,437 up	1,636 up
lmo2378	1.2	2,701 up	1,153 up
lmo2379	1.2	2,109 up	1,158 down
lmo2414	2.2	2,001 up	1,587 up
lmo2418	1.2	2,374 up	1,478 up
lmo2439	5.2	2,416 up	1,861 up

name	Functional category	EGD-e 25°C versus 37°C	125A 25°C versus 37°C
lmo2467	2.1.1	2,792 up	1,627 up
lmo2469	1.2	2,690 up	1,956 up
lmo2473	5.2	2,416 up	1,753 up
lmo2475	2.1.1	2,235 up	1,469 up
lmo2481	3.8	2,237 up	1,867 up
lmo2503	2.4	2,009 up	1,673 up
lmo2515	3.5.2	2,018 up	1,911 up
lmo2517	5.2	2,474 up	1,704 up
lmo2563	5.2	2,816 up	1,300 up
lmo2565	5.2	2,347 up	1,515 up
lmo2569	1.2	2,856 up	1,370 up
lmo2582	1.3	2,336 up	1,856 up
lmo2591	1.8	3,143 up	1,473 up
lmo2641	2.5	2,039 up	1,803 up
lmo2761	2.1.1	2,049 up	1,093 up
lmo2764	2.1.1	2,771 up	1,481 up
lmo2769	1.2	2,434 up	1,084 up
lmo2796	3.5.2	2,255 up	1,363 up
lmo2798	2.1.1	2,831 up	1,681 up
lmo2823	5.2	2,376 up	1,358 up
lmo2832	5.2	3,418 up	1,994 up
lmo2837	1.2	2,197 up	1,913 up
lsp	1.6	2,598 up	1,138 up
lysA		2,692 up	1,454 up
menB	2.5	2,257 up	1,908 up
menD	2.5	2,160 up	1,390 up
menF	2.5	2,149 up	1,216 up
miaA	3.6	2,973 up	1,655 up
minC	1.7	2,149 up	1,000 up
mreB	1.1	2,070 up	1,339 up
mreC	1.1	2,020 up	1,028 down
murF	1.1	2,068 up	1,643 up
mutS	3.2	2,547 up	1,279 up
nusA	3.5.4	3,606 up	1,956 up
parC	3.4	3,250 up	1,408 up
parE	3.4	2,350 up	1,147 up
pduQ	2.1.1	2,020 up	1,999 up
pheS	3.7.2	2,218 up	1,617 up
pheT		2,656 up	1,767 up
plsX	2.4	2,342 up	1,334 up
pnp	2.3	2,221 up	1,744 up
polA	3.1	3,074 up	1,972 up
prfB	3.7.5	2,127 up	1,512 up
proA	2.2	2,461 up	1,343 up
proB	2.2	2,433 up	1,117 up
proS	3.7.2	2,066 up	1,240 up
purB	2.3	2,303 up	1,948 up

name	Functional category	EGD-e 25°C versus 37°C	125A 25°C versus 37°C
purD	2.3	2,159 up	1,925 up
pycA	2.1.2	3,399 up	1,660 up
pyrR	3.5.2	2,279 up	1,080 down
racE	1.1	2,468 up	1,152 up
rbfA	3.7.3	2,707 up	1,513 up
recN	3.3	2,232 up	1,272 up
relA	2.3	2,152 up	1,572 up
rho	3.5.4	2,065 up	1,103 down
rncS	2.3	2,300 up	1,578 up
rplR	3.7.1	2,073 up	1,356 up
rplV	3.7.1	3,247 up	1,573 up
rplX	3.7.1	2,080 up	1,560 up
rpmC	3.7.1	2,243 up	1,662 up
rpsN	3.7.1	2,234 up	1,698 up
rpsQ	3.7.1	2,814 up	1,847 up
rpsS	3.7.1	2,206 up	1,283 up
rsbT	4.1	2,383 up	1,358 up
rsbU	4.1	2,064 up	1,690 up
smbA	2.3	2,339 up	1,504 up
tagB	1.1	3,263 up	1,406 up
tagD	1.1	5,064 up	1,697 up
tcsA	1.2	2,050 up	1,171 up
tktB	2.5	2,579 up	1,266 up
topA	3.4	2,678 up	1,405 up
trmD	3.6	2,428 up	1,623 up
trpB	2.2	3,297 up	1,953 up
trpF	2.2	2,375 up	1,392 up
trpS	3.7.2	2,013 up	1,523 up
truB	3.6	5,427 up	1,750 up
tsf	3.5.3	2,088 up	1,272 up
tyrA	2.2	3,522 up	1,867 up
