# Supplementary material for: Listeria monocytogenes Differential Transcriptome Analysis Reveals Temperature-Dependent Agr Regulation and Suggests Overlaps with Other Regulons
Source: PLoS One. 2012 Sep 14;7(9):e43154. doi: 10.1371/journal.pone.0043154 (PMC3443086; doi:10.1371/journal.pone.0043154)
Supplement: Table S2 — List of genes with higher transcripts levels specifically in L. monocytogenes DG125A at 25°C. (PDF) [file pone.0043154.s003.pdf]

name	Functional category	EGD-e 25°C versus 37°C	125A 25°C versus 37°C
addB	3.3	1,950 up	3,247 up
hisJ	2.2	1,917 up	2,216 up
lmaA	4.5	1,909 up	3,338 up
lmaC	4.5	1,094 down	2,505 up
lmaD	4.5	1,365 down	2,349 up
lmo0051	3.5.2	2,234 down	2,553 up
lmo0056	5.2	1,509 down	2,429 up
lmo0058	6.0	1,999 up	2,359 up
lmo0085	5.1	1,728 up	2,289 up
lmo0106	3.5.2	1,498 up	2,014 up
lmo0148	5.1	1,021 up	2,107 up
lmo0191	2.1.1	1,770 up	2,054 up
lmo0261	2.1.1	1,995 up	2,450 up
lmo0279	2.3	1,103 down	3,290 up
lmo0280	2.3	1,259 up	5,869 up
lmo0293	5.2	1,900 up	2,514 up
lmo0301	1.2	1,727 up	2,572 up
lmo0314	5.2	1,659 up	2,026 up
lmo0368	6.1	1,848 up	2,006 up
lmo0370	5.2	1,497 up	2,045 up
lmo0378	6.0	1,728 up	2,167 up
lmo0381	6.0	1,978 up	4,159 up
lmo0382	3.5.2	1,666 up	2,658 up
lmo0420	5.2	1,843 up	2,027 up
lmo0437	5.2	1,326 up	2,833 up
lmo0476	4.2	1,534 up	2,486 up
lmo0479	5.2	2,405 down	3,709 up
lmo0488	3.5.2	1,740 up	3,173 up
lmo0491	2.2	1,682 up	2,905 up
lmo0504	6.0	1,455 up	3,189 up
lmo0520	3.5.2	1,967 up	3,864 up
lmo0545	6.0	1,427 up	2,032 up
lmo0546	1.4	1,866 up	2,383 up
lmo0552	1.8	1,341 up	2,198 up
lmo0553	5.2	1,437 up	2,239 up
lmo0624	5.2	1,156 up	2,079 up
lmo0625	6.0	1,735 up	2,165 up
lmo0639	3.5.2	1,756 up	2,065 up
lmo0643	2.1.1	1,625 up	4,102 up
lmo0660	4.4	1,582 down	2,759 up
lmo0661	5.2	1,469 up	4,314 up
lmo0672	5.2	1,415 up	2,981 up
lmo0675	6.0	1,671 up	2,145 up
lmo0676	1.5	1,262 up	2,263 up
lmo0677	1.5	1,695 up	3,215 up
lmo0741	3.5.2	1,677 up	2,271 up
lmo0780	6.0	1,778 up	2,914 up

name	Functional category	EGD-e 25°C versus 37°C	125A 25°C versus 37°C
lmo0832	4.4	1,443 up	2,229 up
lmo0851	6.0	1,823 up	2,017 up
lmo0875	1.2	1,721 up	2,374 up
lmo0980	1.2	1,467 up	2,018 up
lmo1006	2.2	1,650 up	2,137 up
lmo1098	4.4	1,886 up	2,129 up
lmo1102	4.2	1,422 up	2,311 up
lmo1133	5.2	1,698 up	2,370 up
lmo1263	3.5.2	1,485 up	3,007 up
lmo1289	1.8	1,554 up	2,238 up
lmo1468	5.2	1,658 up	2,534 up
lmo1529	5.2	1,925 up	2,214 up
lmo1638	5.2	1,259 up	2,093 up
lmo1777	5.2	1,807 up	2,223 up
lmo1780	2.2	1,781 up	3,451 up
lmo1869	5.2	1,853 up	2,396 up
lmo1888	5.2	1,975 up	3,001 up
lmo2049	5.2	1,808 up	2,102 up
lmo2050	3.2	1,192 up	2,036 up
lmo2089	2.4	1,409 up	2,013 up
lmo2145	5.2	1,669 up	2,405 up
lmo2146	3.5.2	1,744 up	3,556 up
lmo2148	5.2	1,632 up	4,030 up
lmo2149	5.2	1,042 down	2,960 up
lmo2151	5.2	1,278 up	2,903 up
lmo2170	5.2	1,110 down	2,685 up
lmo2171	1.2	1,468 up	3,311 up
lmo2173	3.5.2	1,191 up	2,184 up
lmo2204	5.2	1,694 up	2,017 up
lmo2207	5.2	1,893 up	2,098 up
lmo2223	5.2	1,012 down	2,905 up
lmo2238	1.2	1,674 up	2,207 up
lmo2303	4.3	1,684 up	3,075 up
lmo2388	5.2	1,828 up	2,369 up
lmo2446	2.1.1	1,089 down	2,121 up
lmo2470	1.9	1,705 up	2,709 up
lmo2514	5.2	1,802 up	2,064 up
lmo2523	3.1	1,632 up	2,126 up
lmo2577	5.2	1,693 up	2,187 up
lmo2578	5.2	1,351 up	2,126 up
lmo2634	5.2	1,498 up	2,065 up
lmo2642	5.2	1,112 down	2,216 up
lmo2658	2.2	1,794 up	2,083 up
lmo2661	2.1.1	1,370 up	2,265 up
lmo2711	6.0	1,012 down	2,619 up
lmo2749	2.2	1,872 up	2,579 up
lmo2751	1.2	1,947 up	2,807 up

name	Functional category	EGD-e 25°C versus 37°C	125A 25°C versus 37°C
lmo2752	1.2	1,400 up	2,456 up
lmo2753	6.0	1,962 up	2,673 up
lmo2775	1.2	1,861 up	2,292 up
lmo2776	6.0	1,283 up	2,721 up
lmo2807	5.1	1,990 up	2,180 up
lmo2814	3.5.2	1,985 up	2,509 up
lmo2816	1.2	1,668 up	2,723 up
lmo2835	5.2	1,290 up	2,378 up
lmo2836	2.1.1	1,986 up	2,311 up
lmo2841	2.1.1	1,555 up	2,985 up
lmo2842	3.5.2	1,241 up	2,030 up
lmo2846	5.2	1,442 up	3,314 up
recA	3.3	1,110 down	2,189 up
trpE	2.2	1,690 up	2,497 up
