# Supplementary material for: Listeria monocytogenes Differential Transcriptome Analysis Reveals Temperature-Dependent Agr Regulation and Suggests Overlaps with Other Regulons
Source: PLoS One. 2012 Sep 14;7(9):e43154. doi: 10.1371/journal.pone.0043154 (PMC3443086; doi:10.1371/journal.pone.0043154)
Supplement: Table S3 — List of genes with higher transcripts levels specifically in L. monocytogenes EGD-e at 37°C. (PDF) [file pone.0043154.s004.pdf]

name	**Functional category**	**EGDe 37°C vs control 25°C**	**125A 37°C vs control 25°C**
ansB		2,006 up	1,785 up
clpC	4.1	2,430 up	1,999 up
clpP	4.1	2,491 up	1,349 down
cspB	4.1	2,611 up	1,745 up
groEL	3.9	2,610 up	1,703 up
inlC	1.9	2,666 up	1,886 up
lmo0039	2.2	2,388 up	1,639 up
lmo0051	3.5.2	2,234 up	2,553 down
lmo0083	3.5.2	3,806 up	1,543 up
lmo0094	6.0	3,382 up	1,405 up
lmo0102	5.2	2,411 up	1,637 up
lmo0104	6.0	2,084 up	1,619 up
lmo0113	4.3	4,020 up	1,180 up
lmo0114	3.5.2	2,846 up	1,026 down
lmo0155	1.2	2,734 up	1,553 up
lmo0260	5.2	2,347 up	1,381 up
lmo0281	5.1	2,235 up	1,732 up
lmo0295	1.4	2,218 up	1,831 up
lmo0326	3.5.2	3,107 up	1,073 up
lmo0341	5.2	2,964 up	1,112 up
lmo0353	5.2	2,158 up	1,055 down
lmo0421	1.7	2,301 up	1,480 up
lmo0438	6.0	7,127 up	1,841 up
lmo0440	6.0	2,444 up	1,334 up
lmo0465	6.0	2,875 up	1,689 up
lmo0474	6.0	2,103 up	1,842 up
lmo0478	5.2	2,199 up	1,549 down
lmo0479	5.2	2,405 up	3,709 down
lmo0515	5.2	2,783 up	1,011 down
lmo0548	5.1	3,171 up	1,476 up
lmo0579	5.2	2,076 up	1,745 up
lmo0580	2.4	3,027 up	1,984 up
lmo0606	3.5.2	2,176 up	1,453 up
lmo0721	1.8	2,300 up	1,580 up
lmo0731	6.0	3,089 up	1,731 up
lmo0800	5.2	2,457 up	1,598 up
lmo0815	3.5.2	2,002 up	1,468 up
lmo0816	3.5.2	2,192 up	1,731 up
lmo0833	3.5.2	2,215 up	1,758 up
lmo0870	5.2	2,361 up	1,529 up
lmo0871	5.2	2,399 up	1,370 up
lmo0914	1.2	2,679 up	1,073 up
lmo0917	2.1.1	2,050 up	1,223 up
lmo0929	1.8	2,015 up	1,817 up
lmo0996	3.2	2,016 up	1,106 up
lmo1056	6.0	2,708 up	1,726 up
lmo1219	6.0	2,511 up	1,160 down

name	**Functional category**	**EGDe 37°C vs control 25°C**	**125A 37°C vs control 25°C**
lmo1227	3.2	2,494 up	1,410 up
lmo1253	3.5.2	2,245 up	1,645 up
lmo1256	5.2	2,538 up	1,545 up
lmo1257	6.0	3,474 up	1,246 up
lmo1303	5.2	5,782 up	1,984 up
lmo1304	5.2	2,026 up	1,440 up
lmo1333	5.2	3,026 up	1,952 up
lmo1341	1.10	2,216 up	1,029 down
lmo1876	2.5	2,377 up	1,980 up
lmo1975	3.2	2,489 up	1,302 up
lmo2028	5.2	2,034 up	1,816 up
lmo2066	6.0	2,212 up	1,160 down
lmo2175	2.1.1	2,070 up	1,368 up
lmo2187	5.1	2,610 up	1,073 up
lmo2199	5.2	2,427 up	1,614 up
lmo2214	1.2	2,055 up	1,741 up
lmo2252	2.2	2,964 up	1,905 up
lmo2258	6.0	3,589 up	1,437 up
lmo2356	6.0	4,353 up	1,436 up
lmo2364	6.0	3,163 up	1,370 up
lmo2375	6.0	2,648 up	1,101 down
lmo2392	5.2	2,035 up	1,984 up
lmo2396	1.8	2,054 up	1,896 up
lmo2420	6.0	2,220 up	1,621 up
lmo2425	2.2	2,828 up	1,850 up
lmo2432	6.0	2,991 up	1,663 up
lmo2454	6.0	2,422 up	1,074 up
lmo2464	3.5.2	2,198 up	1,673 up
lmo2645	5.2	2,538 up	1,328 up
lmo2651	1.2	2,064 up	1,170 up
lmo2652	3.5.2	2,466 up	1,883 up
lmo2670	5.2	5,632 up	1,982 up
lmo2671	6.0	3,671 up	1,376 up
lmo2675	6.0	3,443 up	1,356 down
lmo2679	1.3	2,123 up	1,518 down
lmo2692	5.2	2,046 up	1,289 up
lmo2701	5.2	2,474 up	1,740 up
lmo2705	5.2	2,231 up	1,581 up
lmo2710	6.0	3,025 up	1,322 up
lmo2721	2.1.1	2,020 up	1,076 down
lmo2722	3.5.2	2,806 up	1,722 up
lmo2723	5.2	2,555 up	1,192 up
lmo2730	2.1.1	4,805 up	1,875 up
lmo2744	3.5.2	2,096 up	1,933 up
lmo2759	5.2	2,466 up	1,516 up
lmo2795	3.5.2	2,540 up	1,444 up
lmo2826	1.2	3,416 up	1,498 up

name	Functional category	EGDe 37°C vs control 25°C	125A 37°C vs control 25°C
lmo2827	3.5.2	3,593 up	1,339 up
lmo2848	2.1.1	2,333 up	1,939 up
ltrC	4.1	2,792 up	1,398 up
plcA	2.4	4,606 up	1,710 up
plcB	2.4	3,798 up	1,675 up
prfA	3.5.2	4,551 up	1,213 up
rpmF		2,365 up	1,626 up
rpsT	3.7.1	2,013 up	1,405 up
sigL	3.5.1	2,020 up	1,592 up
thiD	2.5	2,329 up	1,567 up
