# Supplementary material for: Listeria monocytogenes Differential Transcriptome Analysis Reveals Temperature-Dependent Agr Regulation and Suggests Overlaps with Other Regulons
Source: PLoS One. 2012 Sep 14;7(9):e43154. doi: 10.1371/journal.pone.0043154 (PMC3443086; doi:10.1371/journal.pone.0043154)
Supplement: Table S4 — List of genes with higher transcripts levels specifically in L. monocytogenes DG125A at 37°C. (PDF) [file pone.0043154.s005.pdf]

name	Functional category	EGDe 37°C vs control 25°C	125A 37°C vs control 25°C
accA	2.4	1,320 up	2,378 up
comC	1.10	1,548 up	2,582 up
dltD	1.1	1,475 up	2,615 up
fhuG	1.2	1,120 up	2,355 up
gbuC	1.2	1,257 up	2,015 up
glnR	3.5.2	1,484 up	4,207 up
inlA	1.8	1,252 up	2,167 up
lmo0038	5.2	1,959 up	2,286 up
lmo0091	1.4	1,964 up	2,010 up
lmo0149		1,941 up	2,379 up
lmo0303	6.0	1,273 up	2,056 up
lmo0313	5.2	1,799 up	2,033 up
lmo0331	1.8	1,977 up	2,199 up
lmo0333	1.8	1,350 up	2,090 up
lmo0550	1.8	1,851 up	2,095 up
lmo0590	5.2	1,651 up	2,052 up
lmo0596	5.2	1,929 up	2,811 up
lmo0597	3.5.2	1,419 up	2,215 up
lmo0611	2.1.1	1,958 up	2,420 up
lmo0626	5.2	1,213 up	2,723 up
lmo0645	1.2	1,802 up	2,074 up
lmo0727	2.1.1	1,265 up	2,021 up
lmo0736	2.1.1	1,130 up	3,102 up
lmo0738	1.2	1,052 down	4,799 up
lmo0759	5.2	1,503 up	2,150 up
lmo0761	5.2	1,981 up	2,921 up
lmo0782	1.2	1,189 up	2,170 up
lmo0848	1.2	1,034 up	2,330 up
lmo0866	3.6	1,503 up	2,964 up
lmo0903	5.2	1,974 up	4,471 up
lmo0908	5.2	1,604 up	3,155 up
lmo0920	5.2	1,707 up	2,270 up
lmo0935	3.6	1,174 up	2,161 up
lmo0948	3.5.2	1,924 up	2,356 up
lmo0981	1.2	1,117 up	2,020 up
lmo1013	5.2	1,508 up	2,124 up
lmo1027	5.2	1,729 up	3,247 up
lmo1038	2.5	1,564 up	2,064 up
lmo1079	5.2	1,144 down	2,481 up
lmo1162	6.0	1,340 up	2,023 up
lmo1211	5.2	1,700 up	2,470 up
lmo1261	6.0	1,895 up	2,461 up
lmo1266	5.2	1,704 up	2,363 up
lmo1291	1.1	1,127 up	2,057 up
lmo1375	2.2	1,741 up	3,703 up
lmo1387	2.2	1,770 up	2,059 up
lmo1401	5.2	1,726 up	2,342 up

name	Functional category	EGDe 37°C vs control 25°C	125A 37°C vs control 25°C
lmo1411	5.2	1,275 up	2,199 up
lmo1539	1.2	1,404 up	2,821 up
lmo1549	3.2	1,404 up	2,304 up
lmo1701	6.0	1,965 up	2,122 up
lmo1713	1.1	1,311 up	2,512 up
lmo1738	1.2	1,376 up	3,395 up
lmo1739	1.2	1,108 up	3,318 up
lmo1740	1.2	1,140 up	3,329 up
lmo1751	3.6	1,716 up	2,394 up
lmo1842	5.2	1,971 up	2,109 up
lmo1870	2.6	1,860 up	2,616 up
lmo1910	2.2	1,437 up	2,337 up
lmo1911	5.2	1,385 up	2,126 up
lmo1912	5.2	1,112 down	2,608 up
lmo1914	5.2	1,243 up	2,277 up
lmo1920	5.2	1,692 up	2,304 up
lmo1930	2.5	1,164 up	2,031 up
lmo1978	2.1.1	1,611 up	2,758 up
lmo2015	2.1.1	1,547 up	2,615 up
lmo2045	5.2	1,579 up	2,066 up
lmo2115	1.2	1,499 up	2,100 up
lmo2174	5.2	1,595 up	2,150 up
lmo2184	1.2	1,877 up	2,126 up
lmo2361	5.2	1,871 up	2,112 up
lmo2384	4.1	1,080 down	2,131 up
lmo2423	5.2	1,027 down	2,173 up
lmo2435	5.2	1,509 up	2,004 up
lmo2554	1.1	1,338 up	2,315 up
lmo2772	1.2	1,768 up	2,565 up
lmo2820	3.5.2	1,984 up	2,320 up
murC	1.1	1,636 up	2,284 up
opuCA	1.2	1,339 up	3,378 up
opuCB	1.2	1,418 up	3,963 up
opuCC	1.2	1,313 up	3,354 up
opuCD	1.2	1,593 up	4,809 up
pheT		1,369 up	2,009 up
qoxB	1.4	1,563 up	2,074 up
rpsR	3.7.1	1,631 up	2,327 up
zurM	1.2	1,478 up	3,641 up
