# Supplementary material for: Listeria monocytogenes Differential Transcriptome Analysis Reveals Temperature-Dependent Agr Regulation and Suggests Overlaps with Other Regulons
Source: PLoS One. 2012 Sep 14;7(9):e43154. doi: 10.1371/journal.pone.0043154 (PMC3443086; doi:10.1371/journal.pone.0043154)
Supplement: Table S5 — List of genes with transcripts variations in the analysis DG125A versus EGD-e specific to 25°C. (PDF) [file pone.0043154.s006.pdf]

name	**Functional category**	**125A versus EGD-e at 25°C**
lmo2675	6.0	2,758 up
nadB	2.5	2,668 up
lmo2828	6.0	2,505 up
nadC	2.5	2,380 up
lmo0372	2.1.1	2,369 up
lmo0377	6.0	2,227 up
lmo1190	6.0	2,111 up
lmo2145	5.2	2,067 up
lmo1972	1.2	2,060 up
lmo2308	3.1	2,043 up
nadA	2.5	2,035 up
lmo2324	3.5.2	2,012 up
name	**Functional category**	**125A versus EGD-e at 25°C**
lmo0782	1.2	2,001 down
lmo2230	4.2	2,035 down
lmo0596	5.2	2,056 down
pyrP	1.2	2,112 down
lmo0781	1.2	2,116 down
lmo0628	5.1	2,169 down
opuCA	1.2	2,237 down
opuCB	1.2	2,295 down
inlH	1.8	2,308 down
opuCC	1.2	2,384 down
lmo0019	5.2	2,445 down
lmo0913	2.1.1	2,623 down
lmo0669	2.1.1	2,773 down
lmo0626	5.2	2,799 down
lmo0722	2.1.1	2,817 down
sepA	5.2	2,845 down
opuCD	1.2	2,904 down
lmo1799	1.8	3,070 down
