# Supplementary material for: Listeria monocytogenes Differential Transcriptome Analysis Reveals Temperature-Dependent Agr Regulation and Suggests Overlaps with Other Regulons
Source: PLoS One. 2012 Sep 14;7(9):e43154. doi: 10.1371/journal.pone.0043154 (PMC3443086; doi:10.1371/journal.pone.0043154)
Supplement: Table S6 — List of genes with transcripts variations in the analysis DG125A versus EGD-e specific to 37°C. (PDF) [file pone.0043154.s007.pdf]

| *name* | Functional category | 125A versus EGD-e at 37°C |
|---|---|---|
| *argB* | 2.2 | 2,768 up |
| *argC* | 2.2 | 2,024 up |
| *argD* | 2.2 | 2,123 up |
| *argJ* | 2.2 | 2,219 up |
| *aroB* | 2.2 | 2,111 up |
| *bvrB* | 1.2 | 2,117 up |
| *cbiA* | 2.5 | 2,584 up |
| *cbiF* | 2.5 | 2,128 up |
| *codY* | 3.5.2 | 2,230 up |
| *glnA* | 2.2 | 2,102 up |
| *hisC* | 2.2 | 2,606 up |
| *lmo0003* | 5.2 | 2,145 up |
| *lmo0074* | 5.1 | 2,043 up |
| *lmo0130* | 1.8 | 2,598 up |
| *lmo0132* | 2.3 | 2,246 up |
| *lmo0135* | 1.2 | 2,145 up |
| *lmo0136* | 1.2 | 2,043 up |
| *lmo0138* | 5.1 | 3,701 up |
| *lmo0139* | 5.1 | 2,588 up |
| *lmo0140* | 5.1 | 2,057 up |
| *lmo0142* | 5.1 | 2,113 up |
| *lmo0269* | 1.2 | 2,996 up |
| *lmo0283* | 1.2 | 2,316 up |
| *lmo0284* | 1.2 | 2,892 up |
| *lmo0285* | 1.2 | 2,210 up |
| *lmo0316* | 2.5 | 2,911 up |
| *lmo0344* | 2.1.1 | 2,340 up |
| *lmo0348* | 2.1.1 | 3,078 up |
| *lmo0384* | 2.1.1 | 2,181 up |
| *lmo0456* | 1.2 | 2,124 up |
| *lmo0458* | 2.2 | 2,063 up |
| *lmo0506* | 2.1.1 | 2,660 up |
| *lmo0514* | 1.8 | 2,269 up |
| *lmo0573* | 5.2 | 2,454 up |
| *lmo0598* | 2.5 | 2,809 up |
| *lmo0678* | 1.5 | 4,158 up |
| *lmo0679* | 1.5 | 2,500 up |
| *lmo0683* | 1.5 | 2,244 up |
| *lmo0694* | 6.0 | 2,078 up |
| *lmo0697* | 1.5 | 2,244 up |
| *lmo0699* | 1.5 | 2,703 up |
| *lmo0711* | 1.5 | 2,413 up |
| *lmo0712* | 1.5 | 2,150 up |
| *lmo0735* | 2.1.1 | 3,918 up |
| *lmo0736* | 2.1.1 | 3,721 up |
| *lmo0737* | 5.2 | 3,482 up |
| *lmo0738* | 1.2 | 5,140 up |

| ***name*** | **Functional category** | **125A versus EGD-e at 37°C** |
|---|---|---|
| *lmo0765* | 5.2 | 6,167 up |
| *lmo0766* | 1.2 | 5,944 up |
| *lmo0767* | 1.2 | 8,545 up |
| *lmo0768* | 1.2 | 3,648 up |
| *lmo0769* | 2.1.1 | 4,441 up |
| *lmo0790* | 3.5.2 | 2,061 up |
| *lmo0807* | 1.2 | 2,597 up |
| *lmo0808* | 1.2 | 2,917 up |
| *lmo0809* | 1.2 | 2,253 up |
| *lmo0837* | 1.2 | 2,687 up |
| *lmo0847* | 1.2 | 2,422 up |
| *lmo0862* | 2.1.1 | 2,249 up |
| *lmo0901* | 1.2 | 2,320 up |
| *lmo0903* | 5.2 | 3,157 up |
| *lmo0986* | 1.2 | 2,099 up |
| *lmo0987* | 5.2 | 2,009 up |
| *lmo1031* | 5.2 | 3,420 up |
| *lmo1035* | 1.2 | 2,999 up |
| *lmo1073* | 1.2 | 2,115 up |
| *lmo1091* | 1.1 | 2,090 up |
| *lmo1142* | 2.1.1 | 2,488 up |
| *lmo1143* | 2.1.1 | 2,290 up |
| *lmo1145* | 2.1.1 | 2,092 up |
| *lmo1172* | 3.5.2 | 2,616 up |
| *lmo1173* | 1.3 | 2,493 up |
| *lmo1181* | 2.5 | 2,208 up |
| *lmo1196* | 2.5 | 2,367 up |
| *lmo1207* | 1.2 | 2,116 up |
| *lmo1223* | 1.2 | 2,684 up |
| *lmo1226* | 1.2 | 2,002 up |
| *lmo1239* | 5.2 | 2,975 up |
| *lmo1249* | 5.1 | 2,925 up |
| *lmo1254* | 2.1.1 | 2,242 up |
| *lmo1255* | 1.2 | 2,551 up |
| *lmo1348* | 2.2 | 2,420 up |
| *lmo1349* | 2.2 | 2,807 up |
| *lmo1353* | 5.2 | 2,340 up |
| *lmo1359* | 3.5.4 | 2,023 up |
| *lmo1390* | 1.2 | 2,112 up |
| *lmo1395* | 5.2 | 2,059 up |
| *lmo1416* | 5.2 | 2,228 up |
| *lmo1429* | 5.2 | 2,092 up |
| *lmo1453* | 5.2 | 2,264 up |
| *lmo1492* | 5.2 | 2,273 up |
| *lmo1498* | 4.5 | 2,250 up |
| *lmo1516* | 1.2 | 3,464 up |
| *lmo1517* | 2.2 | 2,924 up |

| *name* | Functional category | 125A versus EGD-e at 37°C |
|---|---|---|
| *lmo1518* | 6.0 | 2,125 up |
| *lmo1537* | 4.5 | 2,048 up |
| *lmo1584* | 5.2 | 2,645 up |
| *lmo1593* | 2.5 | 2,112 up |
| *lmo1625* | 1.2 | 2,270 up |
| *lmo1649* | 5.2 | 2,017 up |
| *lmo1655* | 5.2 | 2,208 up |
| *lmo1662* | 5.2 | 3,016 up |
| *lmo1689* | 3.2 | 2,232 up |
| *lmo1711* | 2.2 | 2,008 up |
| *lmo1729* | 2.1.1 | 2,129 up |
| *lmo1733* | 2.2 | 3,335 up |
| *lmo1734* | 2.2 | 2,491 up |
| *lmo1738* | 1.2 | 2,639 up |
| *lmo1739* | 1.2 | 3,471 up |
| *lmo1740* | 1.2 | 4,187 up |
| *lmo1746* | 1.2 | 2,198 up |
| *lmo1761* | 1.2 | 2,155 up |
| *lmo1810* | 5.2 | 2,301 up |
| *lmo1828* | 5.2 | 2,049 up |
| *lmo1884* | 1.2 | 3,162 up |
| *lmo1885* | 2.3 | 3,045 up |
| *lmo1887* | 5.2 | 2,050 up |
| *lmo1912* | 5.2 | 2,497 up |
| *lmo1926* | 2.2 | 2,697 up |
| *lmo1945* | 5.2 | 2,368 up |
| *lmo1955* | 3.3 | 2,292 up |
| *lmo1970* | 2.4 | 2,168 up |
| *lmo1971* | 1.2 | 2,328 up |
| *lmo1973* | 1.2 | 3,403 up |
| *lmo1999* | 2.1.1 | 2,820 up |
| *lmo2000* | 1.2 | 2,145 up |
| *lmo2003* | 3.5.2 | 2,012 up |
| *lmo2008* | 1.2 | 2,151 up |
| *lmo2104* | 6.0 | 2,898 up |
| *lmo2105* | 1.2 | 2,032 up |
| *lmo2107* | 3.5.2 | 2,122 up |
| *lmo2123* | 1.2 | 2,284 up |
| *lmo2126* | 2.1.1 | 2,573 up |
| *lmo2254* | 5.2 | 2,436 up |
| *lmo2281* | 4.3 | 2,061 up |
| *lmo2282* | 4.3 | 2,522 up |
| *lmo2283* | 4.3 | 2,286 up |
| *lmo2287* | 4.3 | 2,464 up |
| *lmo2289* | 4.3 | 2,005 up |
| *lmo2295* | 4.3 | 2,095 up |
| *lmo2296* | 4.3 | 2,078 up |

| ***name*** | **Functional category** | **125A versus EGD-e at 37°C** |
| --- | --- | --- |
| *lmo2298* | 4.3 | 2,120 up |
| *lmo2299* | 4.3 | 2,054 up |
| *lmo2300* | 4.3 | 2,405 up |
| *lmo2301* | 4.3 | 2,003 up |
| *lmo2316* | 3.2 | 2,353 up |
| *lmo2326* | 4.3 | 2,340 up |
| *lmo2352* | 3.5.2 | 3,969 up |
| *lmo2416* | 5.1 | 2,196 up |
| *lmo2433* | 2.4 | 2,497 up |
| *lmo2563* | 5.2 | 2,192 up |
| *lmo2840* | 2.1.1 | 2,052 up |
| *lsp* | 1.6 | 2,109 up |
| *proB* | 2.2 | 2,353 up |
| *purE* | 2.3 | 2,069 up |
| *pycA* | 2.1.2 | 2,012 up |
| *pyrAB* | 2.3 | 2,729 up |
| *pyrAa* | 2.3 | 3,088 up |
| *pyrB* | 2.3 | 3,653 up |
| *pyrC* | 2.3 | 2,273 up |
| *pyrD* | 2.3 | 3,073 up |
| *pyrDII* | 2.3 | 3,482 up |
| *pyrE* | 2.3 | 2,191 up |
| *pyrF* | 2.3 | 2,779 up |
| *pyrP* | 1.2 | 3,051 up |
| *smc* | 3.4 | 2,007 up |
| *tagD* | 1.1 | 2,365 up |
| *truB* | 3.6 | 2,615 up |
| *tyrA* | 2.2 | 2,109 up |

| ***name*** | **Functional category** | **125A versus EGD-e at 37°C** |
| --- | --- | --- |
| *'comK* | 3.5.2 | 2,049 down |
| *clpE* | 4.1 | 2,133 down |
| *clpP* | 4.1 | 2,652 down |
| *lmo0025* | 2.1.1 | 2,502 down |
| *lmo0026* | 4.2 | 2,913 down |
| *lmo0047* | 5.2 | 2,024 down |
| *lmo0056* | 5.2 | 4,195 down |
| *lmo0083* | 3.5.2 | 2,365 down |
| *lmo0113* | 4.3 | 2,590 down |
| *lmo0144* | 6.0 | 2,954 down |
| *lmo0148* | 5.1 | 2,140 down |
| *lmo0265* | 2.2 | 2,424 down |
| *lmo0279* | 2.3 | 3,094 down |
| *lmo0280* | 2.3 | 3,976 down |
| *lmo0341* | 5.2 | 2,210 down |
| *lmo0438* | 6.0 | 3,665 down |

| *name* | Functional category | 125A versus EGD-e at 37°C |
|---|---|---|
| *lmo0515* | 5.2 | 2,734 down |
| *lmo0602* | 3.5.2 | 2,143 down |
| *lmo0660* | 4.4 | 2,935 down |
| *lmo0670* | 5.2 | 2,934 down |
| *lmo0672* | 5.2 | 2,203 down |
| *lmo0746* | 6.0 | 2,054 down |
| *lmo0780* | 6.0 | 2,364 down |
| *lmo0899* | 5.2 | 2,443 down |
| *lmo0911* | 5.2 | 2,607 down |
| *lmo0914* | 1.2 | 2,018 down |
| *lmo0953* | 6.0 | 2,095 down |
| *lmo0994* | 5.2 | 2,417 down |
| *lmo1050* | 5.2 | 2,048 down |
| *lmo1114* | 4.4 | 2,862 down |
| *lmo1138* | 4.1 | 2,056 down |
| *lmo1219* | 6.0 | 2,060 down |
| *lmo1227* | 3.2 | 3,048 down |
| *lmo1257* | 6.0 | 2,043 down |
| *lmo1341* | 1.10 | 2,611 down |
| *lmo1687* | 5.2 | 2,432 down |
| *lmo1780* | 2.2 | 2,091 down |
| *lmo1868* | 5.2 | 2,005 down |
| *lmo1883* | 2.1.1 | 2,143 down |
| *lmo2131* | 5.1 | 2,587 down |
| *lmo2133* | 2.1.1 | 2,378 down |
| *lmo2149* | 5.2 | 2,064 down |
| *lmo2151* | 5.2 | 2,162 down |
| *lmo2170* | 5.2 | 2,280 down |
| *lmo2223* | 5.2 | 2,961 down |
| *lmo2258* | 6.0 | 2,135 down |
| *lmo2269* | 6.0 | 3,050 down |
| *lmo2356* | 6.0 | 3,100 down |
| *lmo2375* | 6.0 | 2,054 down |
| *lmo2454* | 6.0 | 2,680 down |
| *lmo2511* | 5.2 | 2,747 down |
| *lmo2603* | 5.2 | 2,311 down |
| *lmo2642* | 5.2 | 2,044 down |
| *lmo2686* | 6.0 | 2,253 down |
| *lmo2711* | 6.0 | 2,441 down |
| *lmo2724* | 5.2 | 2,420 down |
| *lmo2792* | 6.0 | 2,013 down |
| *lmo2806* | 5.1 | 2,033 down |
| *lmo2813* | 6.0 | 2,052 down |
| *lmo2828* | 6.0 | 3,483 down |
| *lmo2846* | 5.2 | 2,125 down |
| *lmo2849* | 2.1.1 | 2,166 down |
| *prfA* | 3.5.2 | 3,039 down |

| ***name*** | **Functional category** | **125A versus EGD-e at 37°C** |
| --- | --- | --- |
| *uhpT* | 1.2 | 2,318 down |
