# Supplementary material for: Listeria monocytogenes Differential Transcriptome Analysis Reveals Temperature-Dependent Agr Regulation and Suggests Overlaps with Other Regulons
Source: PLoS One. 2012 Sep 14;7(9):e43154. doi: 10.1371/journal.pone.0043154 (PMC3443086; doi:10.1371/journal.pone.0043154)
Supplement: Table S7 — List of genes with similar trends at 25°C and 37°C in the analysis DG125A versus EGD-e. (PDF) [file pone.0043154.s008.pdf]

name	Functional category	125A versus EGD-e at 25°C	125A versus EGD-e at 37°C
lmo0152	1.2	2,408 up	4,204 up
lmo0345	2.1.1	2,073 up	2,400 up
lmo0346	2.1.2	2,289 up	2,709 up
lmo0347	2.1.1	2,016 up	2,281 up
lmo0349	5.1	2,134 up	4,000 up
lmo0350	5.1	2,128 up	5,978 up
lmo0351	5.2	2,191 up	4,790 up
lmo2143	2.1.1	2,009 up	4,182 up
lmo2257	6.0	5,917 up	3,306 up
lmo2312	6.0	2,067 up	2,030 up
lmo2343	4.2	2,311 up	3,549 up
lmo2344	5.2	2,741 up	3,690 up
lmo2345	5.2	2,906 up	6,216 up
lmo2346	1.2	2,533 up	5,548 up
lmo2347	1.2	3,057 up	7,248 up
lmo2348	1.2	3,320 up	6,944 up
lmo2349	1.2	3,660 up	8,671 up
lmo2350	5.2	3,570 up	11,627 up
lmo2351	1.4	3,926 up	20,247 up

name	Functional category	125A versus EGD-e at 25°C	125A versus EGD-e at 37°C
inlA	1.8	3,464 down	2,001 down
inlB	1.8	2,428 down	2,086 down
lmo0048	1.3	27,690 down	16,239 down
lmo0049	6.0	36,348 down	29,797 down
lmo0050	1.3	10,906 down	11,283 down
lmo0051	3.5.2	5,102 down	29,107 down
lmo0133	5.2	2,206 down	2,192 down
lmo0134	5.2	2,158 down	2,335 down
lmo0302	6.0	4,345 down	3,247 down
lmo0303	6.0	4,020 down	2,488 down
lmo0412	6.0	2,575 down	2,238 down
lmo0477	5.2	21,374 down	44,648 down
lmo0478	5.2	12,316 down	41,967 down
lmo0479	5.2	4,875 down	43,506 down
lmo0778	6.0	2,068 down	6,395 down
lmo0796	5.2	2,014 down	2,268 down
lmo0880	1.8	4,369 down	2,058 down
lmo1241	5.2	2,176 down	2,069 down
lmo1717	5.2	2,587 down	2,546 down
lmo2187	5.1	2,177 down	5,292 down
lmo2669	5.2	2,069 down	2,440 down
lmo2744	3.5.2	2,293 down	2,486 down
